# The global translation profile in a ribosomal protein mutant resembles that of an eIF3 mutant

**DOI:** 10.1186/1741-7007-11-123

**Published:** 2013-12-30

**Authors:** Bayu Sisay Tiruneh, Byung-Hoon Kim, Daniel R Gallie, Bijoyita Roy, Albrecht G von Arnim

**Affiliations:** 1 Department of Biochemistry, Cellular and Molecular Biology, M407 Walters Life Sciences, The University of Tennessee, Knoxville, TN 37996-0840, USA; 2 Department of Natural Science, Albany State University, Albany, GA 31705, USA; 3 Department of Biochemistry, 3432 Boyce Hall, University of California, Riverside, CA 92521, USA; 4 University of Massachussetts Medical School, Worcester, MA 01655-0122, USA; 5 Genome Science and Technology Program, The University of Tennessee, Knoxville, TN 37996, USA

**Keywords:** Translation state, Ribosome occupancy, RPL24, PABP, Regulon, *Arabidopsis*

## Abstract

**Background:**

Genome-wide assays performed in *Arabidopsis* and other organisms have revealed that the translation status of mRNAs responds dramatically to different environmental stresses and genetic lesions in the translation apparatus. To identify additional features of the global landscape of translational control, we used microarray analysis of polysomal as well as non-polysomal mRNAs to examine the defects in translation in a poly(A) binding protein mutant, *pab2 pab8*, as well as in a mutant of a large ribosomal subunit protein, *rpl24b/shortvalve1*.

**Results:**

The mutation of RPL24B stimulated the ribosome occupancy of mRNAs for nuclear encoded ribosomal proteins*.* Detailed analysis yielded new insights into the translational regulon containing the ribosomal protein mRNAs. First, the ribosome occupancy defects in the *rpl24b* mutant partially overlapped with those in a previously analyzed initiation factor mutant, *eif3h*. Second, a group of mRNAs with incomplete coding sequences appeared to be uncoupled from the regulon, since their dependence on RPL24B differed from regular mRNAs. Third, different sister paralogs of the ribosomal proteins differed in their translation state in the wild-type. Some sister paralogs also differed in their response to the *rpl24b* mutation. In contrast to *rpl24b*, the *pab2 pab8* mutant revealed few gene specific translational defects, but a group of seed storage protein mRNAs were stimulated in their ribosome occupancy. In the course of this work, while optimizing the statistical analysis of ribosome occupancy data, we collected 12 biological replicates of translation states from wild-type seedlings. We defined 20% of mRNAs as having a high variance in their translation state. Many of these mRNAs were functionally associated with responses to the environment, suggesting that subtle variation in the environmental conditions is sensed by plants and transduced to affect the translational efficiency of hundreds of mRNAs.

**Conclusions:**

These data represent the first genome-wide analysis of translation in a eukaryote defective in the large ribosomal subunit. RPL24 and eIF3h play similar but non-identical roles in eukaryotic translation. The data also shed light on the fine structure of the regulon of ribosomal protein mRNAs.

## Background

The ribosome is responsible for the translation of all mRNAs into protein. Specific ribosomal protein mutations underlie certain human diseases (ribosomopathies). Different ribosomal protein mutations also cause different spectra of developmental defects in metazoans and plants [1-3]. These observations have raised interest in the contributions of the individual ribosomal proteins to translation of different mRNAs. However, few studies have examined the consequences of ribosome defects on translation, and in the vast majority of case studies no genome-wide data are available. One exception concerns the ribosomal protein of the small subunit, RPS19. A mutation in *Rps19* is responsible for cases of human Diamond-Blackfan anemia. The *rps19* mutation causes both inhibition and stimulation of several dozen mRNAs, including several that are implicated in the etiology of the disease [4]. In another example, a mutation in mouse *Rpl38* results in a homeotic transformation affecting the ribcage. The *rpl38* mutation interferes with translation of homeobox mRNAs [5].

Genome-wide studies of translation under different stresses and environmental conditions have provided a wealth of information on global translation control in the model plant *Arabidopsis thaliana*[6-17]. However, even in *Arabidopsis*, which has become one of the premier model organisms for translatome research, information on the role of specific translation factors is limited. The different isoforms of eukaryotic initiation factors eIF4E and eIF4G, which are components of the mRNA cap-binding complex, contribute differentially to translation [18,19]. The eIF3 complex is the largest of the initiation factors. Some evidence points toward a role for eIF3 in promoting the translation of specific 'client’ mRNAs [20-23]. Only one global plant translation profile has been obtained from a mutant with a defect in the translation apparatus [20]. In detail, mutations that delete the C-terminus of eIF3h compromise translation reinitiation on mRNAs containing upstream open reading frames (uORFs), and also increase the polysome loading of mRNAs with long 5′ leader or coding sequences [20,21,24,25].

Ribosomal protein RPL24 (eukaryote-specific Rpl24e) is a constituent of the cytosolic large 60S subunit. A mutant of *A. thaliana* in which one of three RPL24-encoding genes, *RPL24B*, is deleted (*shortvalve1, stv1*), is characterized by developmental pattern defects in the base of the fruit [26]. Normal fruit patterning is regulated by uORFs and requires RPL24-dependent translation reinitiation on certain uORF-containing mRNAs [25,26]. The major RPL24B paralog, RPL24A, has also been implicated in the translation reinitiation on mRNAs with multiple coding regions [22]. In single-gene reporter gene expression assays of the uORF-containing mRNA, *AtbZIP11*, the molecular defects in *rpl24b* resemble those in the *eif3h* mutant. The seedling phenotypes of *eif3h* and *rpl24b*, while not identical, overlap with respect to vascular development, gynoecium structures, and other organs [25]. These data suggest that RPL24B and eIF3h have related functions, although their exact biochemical roles are unclear.

In *Arabidopsis*, all ribosomal proteins are encoded by at least two paralogs, which are typically at least partially redundant [1,27]. *RPL24A* (At2g36620) and *RPL24B* (At3g53020) are expressed at similarly high levels. The third and final paralog, *RPL24C* (At2g44860), is expressed at a fourfold lower level, and the protein may be enriched in the nucleolus [28]. RPL24 has also been investigated in mouse and yeast. A mutation in the single mouse *Rpl24* gene, *Belly spot and tail* (*Bst*), is homozygous lethal while heterozygotes show a pleiotropic developmental phenotype [29]. For comparison, a double mutant in the two *RPL24* genes of the yeast *Saccharomyces cerevisiae* is not lethal [30,31]. These results suggest that the yeast ribosome can assemble successfully without RPL24, in keeping with RPL24 being one of the last proteins to be assembled into the 60S subunit [32].

Another specific protein that has been implicated in plant translational control is poly(A)-binding protein (PABP) [33-36]. By bridging between the 5′ cap binding complex and the 3′ poly(A) tail, PABP is thought to enhance ribosome recycling and thus translation [37-39]. In *Arabidopsis* the class II *PABP* genes (*PAB2*, *PAB4* and *PAB8*) are the more highly expressed in seedlings and encode the bulk of PABP in seedlings [33]. There are no reports of mRNA-specific translational defects for any *pab* mutant.

For this study, we sought to identify the translational defects in two new types of mutants that affect the translation apparatus. We report here one of the first global analyses of ribosome occupancy in any organism for a ribosomal protein mutant, *Arabidopsis rpl24b*. This mutant was chosen because of its functional association with the initiation factor eIF3h, for which a mutant translatome is already available [20]. We also report ribosome occupancy data in the *Arabidopsis pab2 pab8* double mutant. This mutant is viable, yet slightly dwarfed, indicative of compromised PABP activity; a *pab2 pab4 pab8* triple mutant has not been recovered [34]. The translational defects in the *pab* mutant were comparatively subtle and restricted to residual mRNAs that are expressed highly in late embryogenesis. In contrast, the *rpl24b* mutation altered the ribosome occupancy of hundreds of mRNAs. In keeping with the related developmental phenotypes of the *rpl24b* and *eif3h* mutations, their translational defects also overlapped. Ribosomal protein (r-protein) mRNAs were the most highly affected functional category. The ribosome occupancy of nuclear encoded r-proteins was typically stimulated by the *rpl24b* mutation. A large fraction of r-proteins, but not all, appear to belong to a single regulon of translational control.

## Results

### Identification of differentially translated genes in a ribosomal protein mutant

**Figure 1:**
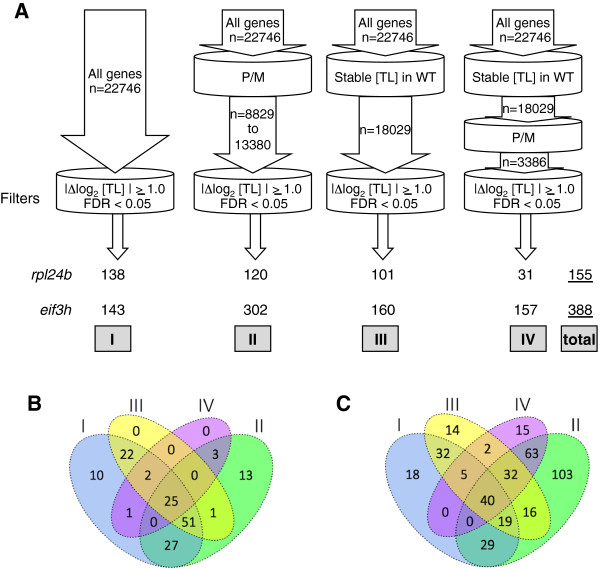
**Differentially translated genes in *rpl24b *and *eif3h *mutant *Arabidopsis *seedlings. (A)** A total of 155 and 388 non-redundant genes with differential translation state (TL) (|Δlog_2_ translation state| ≥1.0, false discovery rate (FDR) <0.05) were identified in *rpl24b* and *eif3h* seedlings, respectively. The Roman numerals represent the four gene sets used in identifying differentially translated genes (DTGs), and three of the sets were first filtered by one or more methods. I = all *Arabidopsis* genes (n = 22,746) represented on GeneChip® ATH1 Genome Array; II = all genes with raw signal values detected above background or no 'A’ calls in at least 50% of arrays (n = 8 to 12); III = all genes with stable translation state (TL) in 12 wild-type 'replicate’ samples (n = 18,029 genes); IV = genes from set III that had a 'P/M’ call for each of 24 replicate arrays from wild-type seedlings. The number of DTGs from each of the gene sets is shown at the bottom, and the number in the non-redundant set is underlined on the right (total). **(B,C)** Venn diagrams showing the overlap in the number of DTGs from the four gene sets in **(A)** for *rpl24b***(B)** and *eif3h***(C)** datasets. The rate of false positives (FDR) in the total non-redundant set will be slightly higher than 0.05, due to the increase in the number of comparisons.

Seedlings of previously described mutant alleles were grown for the *pab2 pab8* double mutant [34], the *eif3h-1* mutant [21], and *rpl24b* (*shortvalve1-1*) [26] (see Methods). Polysome microarray data from the *rpl24b* mutant and the *pab2 pab8* mutant were collected using sucrose gradient fractionation of ribonucleoprotein complexes from *Arabidopsis* seedlings and were processed as described in Methods. In parallel, equivalent data from a published experiment [20] on the *eif3h* mutant were reanalyzed from the hybridization signals using the same procedure as for *rpl24b*. The translation state (TL) is defined as the ratio between the mRNA signal in the polysomal: non-polysomal fractions and is displayed as a log_2_ value. For example, an mRNA that is 80% polysomal and 20% non-polysomal receives a TL of 4.0 and a log_2_ TL of +2.0. For this reason the TL value is completely independent of the transcript level (TC). We carefully evaluated many different data processing procedures in order to optimize detection of differentially translated genes (DTGs; see Methods). DTGs between mutant and wild-type were first mined using a stringent false-discovery-rate (FDR) cut-off of 5%. From the entire set of 22,746 probe sets, we identified 138 and 143 DTGs for *rpl24b* and *eif3h*, respectively. Because this number for *eif3h* was small compared to the number of DTGs identified in the earlier publication [20], we sought to expand the gene set. Using the 3 additional prefiltering methods laid out in the Methods, we assembled a non-redundant set of DTGs, 155 for *rpl24b* and 388 for *eif3h* (Figure 1A-C).

### Comparison of translation defects between *rpl24b* and *eif3h*

Because translation of *AtbZIP11*, *AUXIN RESPONSE FACTOR3* (*ETTIN*) and *ARF5* (*MONOPTEROS*) depend on functional eIF3h and RPL24B [25,26], one might hypothesize that eIF3h and RPL24B control the ribosome loading of identical sets of client mRNAs. We tested this hypothesis by comparing clusters of genes that are altered at the translation level in *eif3h* and *rpl24b* mutants.

**Figure 2:**
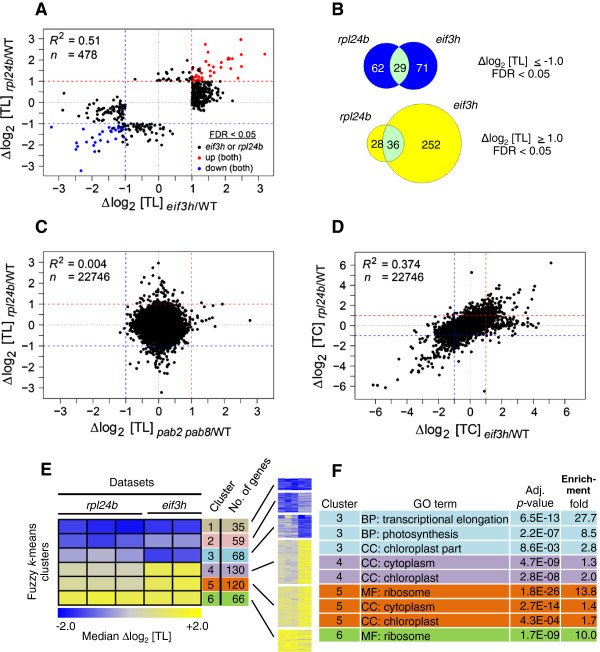
**Translation states (TL) in the *rpl24b *mutant resemble those in *eif3h*. (A)** Scatterplot comparing translational alterations in the *rpl24b* mutant with those in the *eif3h* mutant. Only differentially translated genes (DTGs), that is, genes with differential translation state (|log_2_ ΔTL| ≥1.0, false discovery rate (FDR) <0.05; n = 478) are shown. Blue, differentially undertranslated in both mutants; red, differentially overtranslated in both mutants; black, differentially translated in either mutant. R^2^, Pearson correlation coefficients. Dashed lines represent twofold changes. **(B)** Venn diagrams showing the translational coregulation of sets of mRNAs by RPL24B and/or eIF3h. Overlap for genes undertranslated (top**)** or overtranslated (bottom) in the *eif3h* and/or *rpl24b* mutant. **(C)** Scatterplot comparing translational alterations in the *rpl24b* mutant with *pab2 pab8* mutant. All genes on the ATH1 array are shown. **(D)** Scatterplot comparing total mRNA transcript levels (TC) between *rpl24b* and *eif3h*. **(E)** Heatmaps of fuzzy k-means clustering of 478 differentially translated genes identified from Robust Multi-array Average-normalized combined data of *rpl24b* (3 biological replicates) and *eif3h* mutants (2 replicates). The heatmaps show median Δlog_2_ TL expression values for each of six clusters of mRNAs that show similar translation defects in each mutant over wild-type. A TL = 2.0 corresponds to 80% of the mRNA in the polysomal fraction, and TL = -2.0 corresponds to 20% in the polysomal fraction. The number of genes in each cluster is indicated. The heatmap color panel indicates undertranslation (blue), overtranslation (yellow) and no change (white) in mRNA translation. **(F)** List of the most significantly enriched Gene Ontology (GO) categories for each cluster. GO terms, BP: biological process; CC: cellular component; MF: molecular function. Adjusted *P* values and the fold enrichment of the given GO category compared to the reference set are listed.

Among the DTGs in *rpl24b* or *eif3h*, the direction and the degree of mistranslation were significantly correlated (R^2^ = 0.51). This indicates that translation of many mRNAs are coregulated by, or codependent on, RPL24B and eIF3h (Figure 2A,B). Remarkably, among all the mRNAs that were overtranslated in *eif3h (*Δlog_2_ TL >1, FDR <0.05), the vast majority had a trend towards overtranslation in the *rpl24b* dataset (Δlog_2_ TL >0; *P* <0.0001, ranked sign test); the same held true for undertranslated mRNAs (*P* <0.0001). This result indicates that a substantial and significant number of *Arabidopsis* mRNAs are codependent on eIF3h and RPL24B for appropriate translation. The mRNAs that were translationally stimulated in both *rpl24b* and *eif3h* mutants were enriched in ribosomal protein mRNAs (Additional file 1, also below) while the undertranslated ones had no detectable functional bias.

The effects of *rpl24b* and *eif3h* on transcript abundance were also correlated (R^2^ = 0.374) (see Figure 2D; Additional file 1). Because RPL24 and eIF3h are translation factors, we presume that most of the changes at the transcript level are a consequence, rather than a cause, of alterations in translation. We also note that the translational defects in the *rpl24b* mutant are not strongly correlated with changes in total transcript levels, similar as for *eif3h*[20] (Additional file 2: Figure S1).

To work out whether there are mRNAs that depend on only 1 of the 2 genes, *RPL24B* or *eIF3h*, but not on the other, we performed fuzzy *k*-means clustering on a total of 478 DTGs (Figure 2E). Two of six clusters, clusters 1 and 6, corresponded to the strongly coregulated mRNAs. Two other clusters, clusters 2 and 5, showed more limited coregulation, that is, a strong response in one mutant and a moderate trend in the other. The last two clusters, clusters 3 and 4, contained mRNAs whose response was much stronger in *eif3h* than in *rpl24b*. There were few *RPL24B*-specific mRNAs.

Overall, these results underscore the significant overlap between sets of mRNAs mistranslated in *eif3h* and *rpl24b* mutants. These new findings bolster the conclusion that *RPL24B* and *eIF3h* play related roles in eukaryotic gene expression.

### Translation states in the *pab2 pab8* double mutant

**Figure 3:**
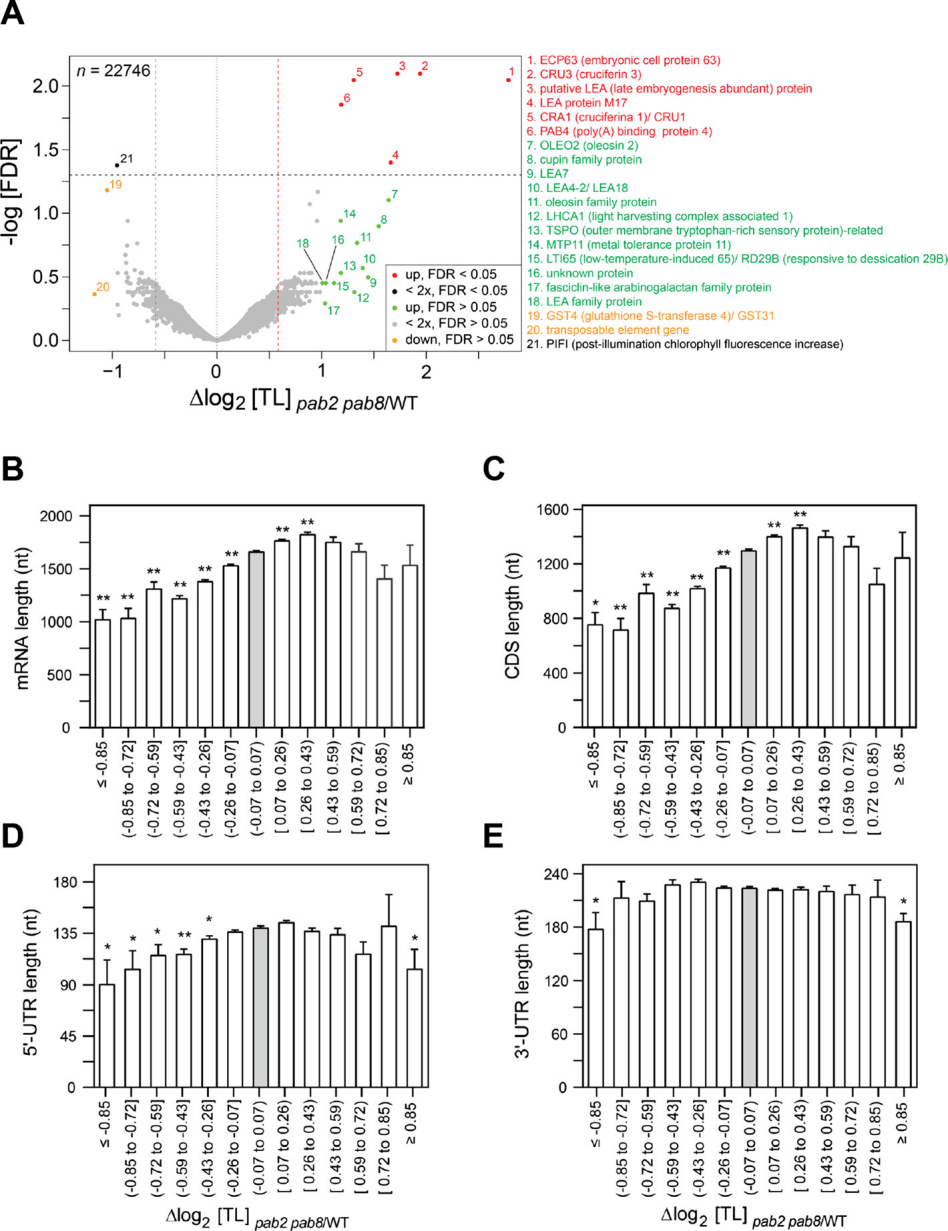
**Translation states (TL) in the *pab2 pab8 *poly(A) binding protein mutant.** Δlog_2_ TL indicates translational stimulation (>0) or inhibition (<0) in the *pab2 pab8* mutant as compared to wild-type. **(A)** Volcano plot showing the distribution of alterations in ribosome occupancy (x-axis) versus the false discovery rate (FDR, y-axis). Many of the translationally stimulated mRNAs encode seed storage proteins. The color coding distinguishes mRNAs with FDR-validated significance (red for upregulated, black for downregulated) from mRNAs showing trends only (green for upregulated, orange for downregulated). Genes above the stippled line are less than 5% likely to be false positives. **(B-E)** The effect of the *pab* mutation on ribosome occupancy is related to mRNA length **(B)**, specifically the lengths of the coding sequence **(C)** and 5′ untranslated region (UTR) **(D)**, rather than the 3′ UTR **(E)**. Asterisks indicate a significant difference compared to the gray 'no change’ bin (Student t test); **P <0.0001; * ≥ 0.0001 *P* <0.05. Error bars are standard errors of the mean.

The poly(A) binding proteins are widely thought to stimulate translation in a cellular context by helping to juxtapose 3′ end and 5′ end of mRNAs and by assisting with ribosome recycling. *PAB2*, *PAB4*, and *PAB8* are the three major PABP genes expressed in seedlings and are functionally redundant. The *pab2 pab8* double mutant is mildly dwarfed [33,34]. We examined the ribosome occupancy of mRNAs in the *pab2 pab8* double mutant seedlings (Figure 3). Overall, the *pab2 pab8* double mutant had few gene-specific alterations in its ribosome occupancy. The primary FDR-controlled alteration was an approximately twofold increase in ribosome occupancy for a small group of seed storage protein mRNAs (Figure 3A). This result is of interest, as seed storage protein mRNAs are under translational control in various other plants [40]. Less stringent filtering using an alternative *ad hoc* method to identify DTGs (see Methods) recovered oleosins, which are additional seed storage mRNAs, and Late Embryogenesis Abundant mRNAs among the mRNAs with increased ribosome loading in *pab2 pab8*. It is notable that *PAB4*, the major remaining *PABP* mRNA in the *pab2 pab8* mutant, was also translationally stimulated. This result favors the hypothesis that *PABP* mRNAs are subject to negative translational autoregulation via poly(A) stretches in their 5′ untranslated regions (UTRs) [33].

We compared the translational alterations in the *rpl24b* mutant with those in *pab2 pab8*. Not even a weak correlation was detected (Figure 2C). This important result suggests that different perturbations of the translational machinery affect different sets of mRNAs.

Because most alterations in ribosome occupancy in *pab2 pab8* were small and statistically insignificant on a single-gene basis, we searched for global patterns. Short mRNAs were enriched among the mRNAs that were translationally inhibited in the *pab2 pab8* mutant (Figure 3B). This trend could be attributed to the length of the coding sequence as well as the length of the 5′ UTR, while the length of the 3′ UTR contributed little if at all (Figure 3C-E).

Analysis of mRNA transcript levels in the *pab2 pab8* mutant revealed 24 and 107 significantly downregulated and upregulated genes at the mRNA transcript level. The transcript levels of *PAB2* and *PAB8* were reduced, as expected given the T-DNA insertions in these genes in the *pab2 pab8* strain. Interestingly, among the 14 mRNAs with >2-fold increased ribosome occupancy in *pab2 pab8* all except 1 (*PAB4*) are late-embryogenesis mRNAs whose level declines up to 1000-fold during seed germination, the developmental period preceding our analysis. Of them, 12 were also upregulated at the transcript level in *pab2 pab8*. For *pab2 pab8*, and in contrast to the situation in *rpl24b*, the correlation between translational and transcript-level stimulation continued among the less strongly stimulated mRNAs (Additional file 1). This result suggests that wild-type PABP might couple transcript turnover and translation state for these late embryogenesis mRNAs.

### Overtranslation of ribosomal protein mRNAs in *rpl24b* and *eif3h*

We performed genome ontology (GO) analyses of the six clusters of RPL24B or eIF3h dependent mRNAs identified by fuzzy k-means clustering (Figure 2E,F). Clusters 6 and 5 were both highly enriched for r-protein mRNAs. Of note, cytosolic r-protein mRNAs are coordinately affected in response to sucrose starvation [13], drought [9], hypoxia stress [6,7], and unanticipated darkness [8]. However, in each of these stresses, the r-protein mRNAs are undertranslated rather than overtranslated.

To detect smaller effects of RPL24B and eIF3h on the ribosomal protein mRNAs, we adopted the *ad hoc* filtering method to identify differentially translated genes (DTG^adhoc^) [20]. The *ad hoc* method does not control for FDR (see Methods). This method enriched even more significantly for r-proteins (*P* = 4.8E-03 to 2.1E-68; Additional file 3: Figure S2). Of all the cytosolic r-protein mRNAs for which the arrays yielded data (n = 137), 41% were found in clusters 4 to 7, a 5.8-fold enrichment. This result suggests that the *ad hoc* method, which is less conservative than the FDR method, produces fewer false negative calls. These results, therefore, confirm in more detail that many but not all cytosolic r-protein mRNAs depend on RPL24B and eIF3h at the translation level and could define a regulon of translational control [20]. mRNAs that were affected by *eif3h* or *rpl24b* mutations were not generally perturbed by the *pab2 pab8* mutations or by a mutation in the k subunit of eIF3 (*eif3k*) (Additional file 3: Figure S2C).

**Figure 4:**
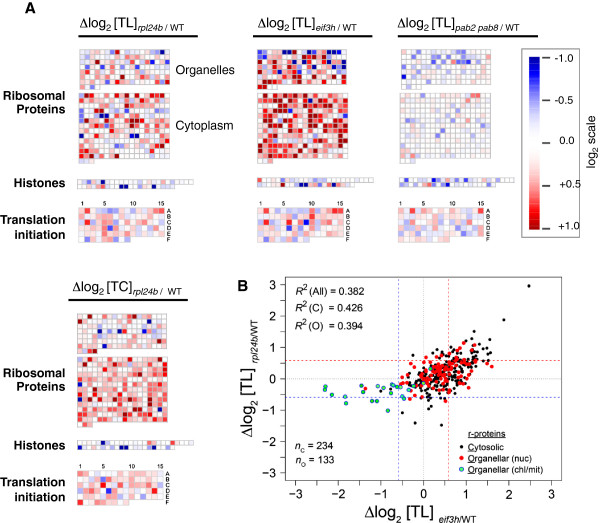
**Enhancement of translation of ribosomal protein mRNAs in the *rpl24b *mutant. (A)** Changes in translation state (Δlog_2_ TL) were plotted onto *Arabidopsis* biochemical pathways and functional categories using MapMan v3.5.1R2. Each square represents a single gene, and each gene occupies equivalent positions in each set. The log-scale indicates overtranslation (red) or undertranslation (blue) in *rpl24b* or *eif3h* or *pab2 pab8* compared to wild-type. Ribosome occupancy of ribosomal protein mRNAs is stimulated in *rpl24b*, which is enhanced in *eif3h* (middle). The similarity does not extend to histones, which are shown as a representative comparison group. It also does not extend to *pab2 pab8*. Also note that transcript levels (Δlog_2_ TC _*rpl24b/WT*_) for cytosolic r-proteins tend to be elevated in the *rpl24b* mutant. Data are for selected gene classes from all *A. thaliana* probe sets (genes) on the GeneChip® Arabidopsis ATH1 Genome Array (n = 22,746). **(B)** Scatterplot showing the translational codependence of cytosolic (black filled circles) and organellar (red and green filled circles) r-protein mRNAs on RPL24B and eIF3h. Genes encoding organellar r-protein mRNAs are further subdivided into nuclear-encoded (nuc) and chloroplast-encoded or mitochondrion-encoded (chl/mit). Pearson correlation coefficients (R^2^) for cytosolic (C), organellar (O) and all (All) r-protein mRNAs are indicated. Dashed lines (blue and red) represent 1.5-fold changes. The number of mRNAs (n) for each class of r-protein is also indicated.

We next visualized the r-protein cohort at a gene-specific level (Figure 4A,B). Among mRNAs encoding cytosolic r-proteins the pattern of overtranslation in the *rpl24b* mutant was generally mirrored and enhanced in the *eif3h* mutant. In contrast, the pattern in the *pab2 pab8* mutant bore no resemblance to that in *rpl24b* or *eif3h*. The joint overtranslation in *rpl24b* and *eif3h* did not generally extend to the translation initiation factors; among the few that were jointly stimulated, we found a few eIF3 subunits (Figure 4A; cell 6C, eIF3g; 2A, eIF3i; 14A, eIF3k). The mutation in *rpl24b* also stimulated the mRNA transcript levels of many cytosolic r-protein mRNAs, but the changes at the transcript level were not obviously correlated with those at the translation level.

Among the mRNAs for organellar r-proteins, those encoded in the nucleus were translationally stimulated in a similar fashion in the two mutants, although the trend was weaker than for the nuclear-encoded cytosolic proteins (Figure 4B, Additional file 4: Figure S3). In contrast, mRNAs for r-proteins that are encoded in the organelles themselves, that is, the plastid or mitochondrion, all had reduced ribosome loading in the *rpl24b* and *eif3h* mutants. The effect on the organellar mRNAs must be indirect because RPL24 and eIF3h function only in the cytosol. It should be noted that our array data are biased for polyadenylated mRNAs. In the organelles, polyadenylation is a precursor for degradation [41]. Thus, our results suggest that, in the wild-type chloroplasts, polyadenylated mRNAs remain substantially ribosome loaded, whereas in the mutants, the ribosome loading of the polyadenylated mRNAs is reduced.

### Fine structure analysis of the translation status of ribosomal protein mRNAs

**Figure 5:**
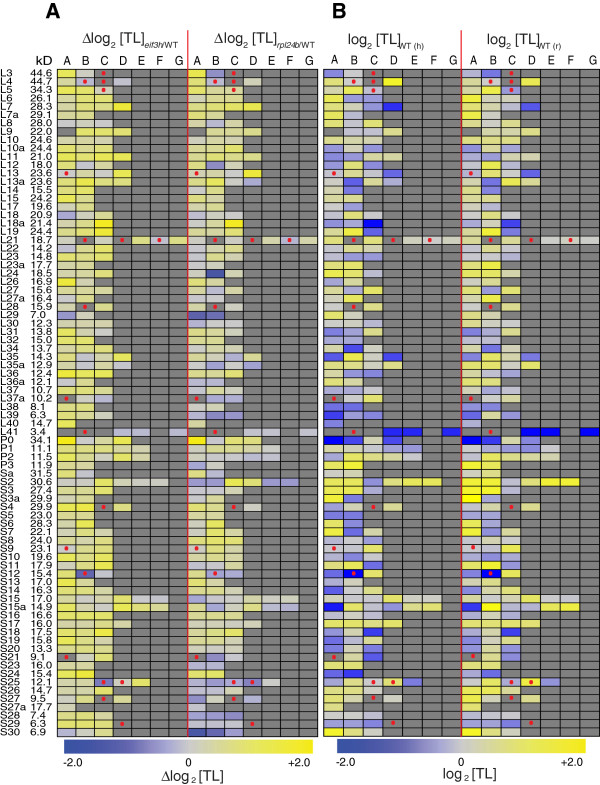
**Translation states of ribosomal protein paralogs.** Each ribosomal protein is encoded by up to seven paralogs named A to G. The molecular weight of the protein is indicated on the left. The heatmap in **(B)** shows absolute ribosome occupancy of the r-protein mRNA in wild-type plants, while the heatmap in **(A)** shows the increase (yellow) or decrease (blue) in ribosome occupancy in the *rpl24b* and *eif3h* mutants (see scale at the bottom). Gray color indicates that the gene does not exist or is not represented on the array. Dots indicate pseudogenes and incomplete open reading frames (ORFs). In **(B)**, the left and right panels show average ribosome occupancy (Translation State) from two replicates of the wild-type control plants grown for the *eif3h* experiment [20] and from three replicates of the wild-type of the *rpl24b* experiment (this study), respectively.

Because the r-protein mRNAs were reliably expressed and showed robust cotranslation among themselves and between *rpl24b* and *eif3h* mutants, we characterized their translational coregulation in greater detail. Figure 5B shows that the ribosome occupancy (Translation State) of the r-protein mRNAs in wild-type plants varied dramatically among the different mRNAs. Certain extremely short mRNAs should have low ribosome occupancy, simply because few ribosomes fit onto the coding sequence of a small mRNA. This was indeed the case for several of the smallest r-proteins (for example, L41). However, even among the paralogs of one family, it was common for one paralog to be highly translated, and another paralog to be poorly translated (for example, L7, S12). However, the reproducibility of the ribosome occupancy between the two replicate experiments was excellent. Certain r-protein mRNAs have incomplete coding sequences [42] and have been marked as pseudogenes [43]. These mRNAs tended to have lower ribosome occupancy (Figure 5B), Additional file 5.

Figure 5A shows how ribosome occupancy of the r-protein mRNAs was affected in the *rpl24b* and *eif3h* mutants. The following numbers of mRNAs cleared statistical significance at FDR <0.05: 42 mRNAs that changed ribosome loading in *eif3h* and 16 in *rpl24b* (Figure 5A and Additional file 4: Figure S3 and Additional files 1 and 5). Several generalizations are supported by these data. First, certain mRNAs with very high ribosome occupancy in the wild-type (Figure 5B) did not increase their TL in the mutant, presumably simply because TL was already close to maximal (for example, L4D and S3aA). Vice versa, mRNAs with medium or low ribosome occupancy in the wild-type had an upward trend in the mutant (for example, L7D, L18aC). Second, for many r-protein families, the degree of translational dependence differed between the different paralogs; clear examples include L3, L12, L13, L18a, L21, L35a, P0, Sa, S2, S12, S15a, S25, and S30. Third, in keeping with the pattern of cotranslation between *rpl24b* and *eif3h* described earlier (Figure 3A) the paralog with the strongest stimulation in *rpl24b* often had the strongest stimulation in *eif3h*. For examples, see L3, L4, L7, L12, L13, L18a, L21, L22, L26, L29, P0, S6, S18, S21, and S30. Clear exceptions were rare. RPL24 presents one of the few exceptions, almost certainly because the *RPL24B* mRNA is truncated by a deletion in the *rpl24b* mutant [26].

Fourth, in a minority of r-protein families all paralogs were slightly reduced in their ribosome loading in the *rpl24b* mutant. Most of these r-proteins were small proteins of less than 10 to 13 kDa [42]. Examples are L29 (7 kDa), L35a (12.8 kDa), L36a (12.1 kDa), L37a (10 kDa), L39 (6.4 kDa), S21 (9 kDa), S25 (12.1 kDa), S27 (9.5 kDa), S28 (7.4 kDa), and S30 (6.9 kDa). In contrast, most of the larger r-proteins had at least one paralog that was translationally stimulated in the *rpl24b* mutant. On this topic, we note that the translation of these short mRNAs was generally normal in the *eif3h* mutant. In the *eif3h* mutant, long mRNAs are preferentially inhibited in their ribosome loading, but short mRNAs are not [20]. This is one exception from the general rule of correlated translation behavior between the two mutants.

Finally, certain r-protein paralogs code for incomplete open reading frames. Of the eight such cases [42] for which we have data (Figure 5A), none were translationally stimulated in either of the mutants, in sharp contrast to the general pattern. Indeed, the two mRNAs with the strongest translational repression in *eif3h*, *RPS12B* and *RPS25C*, both encode incomplete ORFs and are annotated as pseudogenes in The Arabidopsis Information Resource, release 10. It is unclear why these mRNAs are more sensitive to the mutations.

Taken together, considering the detailed annotation features just discussed, it appears that the r-protein regulon probably includes more mRNAs than was evident from Figure 4. While Figure 4 suggested that certain r-protein mRNAs might be excluded from the regulon, detailed inspection of Figure 5 makes this hypothesis harder to sustain. Of the cytosolic r-proteins above 13 kDa, the majority showed translational stimulation in both *rpl24b* and *eif3h* for at least one paralog. Paralogs that were stimulated less in the mutants often had a near maximal translation state in the wild-type. These mRNAs should be counted as legitimate members of the r-protein regulon. Other paralogs that bucked the general pattern and should not be considered part of the r-protein regulon code for incomplete r-proteins and *RPL24B* itself.

### Molecular features of undertranslated and overtranslated mRNAs

Given that the *rpl24b* mutant shared a common set of translationally stimulated and translationally inhibited mRNAs with *eif3h*, we examined whether these mRNAs harbored the same features that render some *Arabidopsis* mRNAs *eIF3h*-dependent. These features are uORFs and a long coding region [20]. As shown in Additional file 6: Figure S4, uORFs only played a weak role in causing poor translation in *rpl24b*. In detail, a statistically significant association between uORFs and *RPL24B*-dependent translation was seen only within the middle range of the distribution (> - 0.6 ΔTL <0.8), but not for the more strongly *RPL24B*-dependent mRNAs (ΔTL < -0.6). The association of ORF length with *RPL24B*-dependence was also weak (Additional file 6: Figure S4A). In contrast, for the *eif3h* dataset, our reanalysis of the earlier data [20] reproduced the conclusion that uORFs correlate with eIF3h dependence (Additional file 6: Figure S4B). To get a clearer view of this unexpected result, we examined the occurrence of uORFs among the undertranslated genes found in clusters 1, 2, and 3 (Figure 2). The mRNAs that were clearly codependent on both *RPL24B* and *eIF3h*, were not enriched for uORFs (Additional file 6: Figure S4C), while the mRNAs that were strongly *eIF3h*-dependent but weakly *RPL24B*-dependent tended to be the ones with uORFs. Apparently, the *rpl24b* mutation caused only a minor deficiency in ribosome loading for the uORF-containing mRNAs. This was surprising, because if one measures reporter protein translation downstream from a uORF in the *rpl24b* mutant, the deficiency in translation can be quite pronounced [25,26]. In summary, the effects of the mutations in *eIF3h* and *RPL24B* on ribosome occupancy are related but not identical.

### Stochastic variation of mRNA translation states in wild-type plants

**Figure 6:**
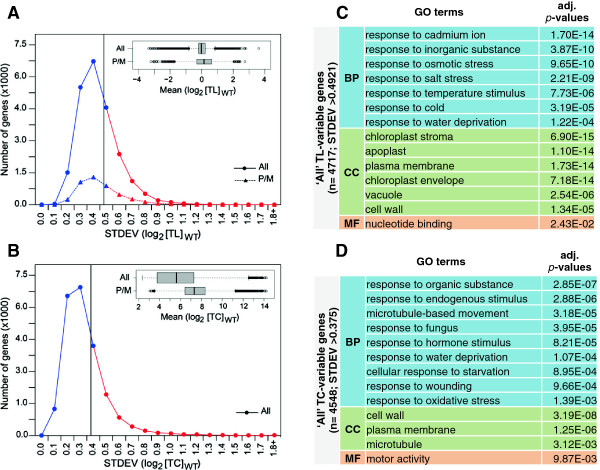
**Variability of translation states in wild-type plants under controlled growth conditions.** Translation state (TL) values and transcript levels (TC) were collected from 12 *bona fide* identical biological replicates of wild-type seedlings grown under standard growth conditions. **(A)** Line histogram showing the distribution of the standard deviations (SD) of the translation state (TL). The 20% of mRNAs with the highest standard deviation (>0.49) are highlighted in red to the right of the vertical line. The inset shows the distribution of the TL values as a boxplot. **(B)** Line histogram showing the distribution of standard deviations for total transcript levels. The 20% of mRNAs with the highest standard deviation (>0.375) are highlighted in red to the right of the vertical line. The inset shows the distribution of total transcript levels as a boxplot. **(C)** GO analysis of mRNAs with highly variable translation state using the DAVID (Database for Annotation, Visualization and Integrated Discovery) functional annotation tool. **(D)** GO analysis of mRNAs with highly variable transcript levels.

Data on mRNA translation states can be variable between biological replicates. We examined the structure of the variation in translation states that we observed among 12 different RNA samples from wild-type seedlings, collected from seedlings grown on defined agar medium under largely identical experimental conditions. We focused on the 20% of mRNAs with the largest variation in translation state among the replicates, that is, a standard deviation above 0.49 (Figure 6A). Likewise, we identified a group of mRNAs with highly variable mRNA transcript levels (Figure 6B).

Gene ontology (GO) of the translationally variable mRNAs revealed that the most enriched biological process (BP) was 'response to abiotic stimulus’. Within this category, many different stimuli and stresses were represented and none stood out (Figure 6C). The most enriched cellular compartment was the 'chloroplast’ , and multiple compartments such as 'stroma’ and 'envelope’ , but not 'thylakoid’ , were overrepresented. The overrepresentation of plasma membrane, apoplast, cell wall, and vacuole suggests an abundance of secretory mRNAs.

The functional bias of the translationally variable genes towards 'abiotic stimuli’ suggests that the variation in translation state is likely a deliberate response of *Arabidopsis* to subtle differences in growth conditions. This was the case even though all four experiments were performed by the same person in the same growth chamber under nearly identical conditions. Given the diversity of stimuli listed, several different environmental factors may be involved. This result is also in keeping with the notion that many mRNAs that are functionally associated with environmental plasticity are regulated at the translational level [6-11,13,15,16]. We suggest that noticeable effects on translation state can occur in response to very subtle (that is, unintentional) differences in growth conditions.

## Discussion

This study describes the first global analysis of defects in ribosome loading in a plant ribosomal protein mutant and one of few such studies in eukaryotes [4]. The cytosolic RPL24, a subunit of the large (60S) subunit of the ribosome, is located near the 40S to 60S subunit interface on the lower hemisphere of the 60S, below the elongation factor binding site. During translation elongation, the protein faces forward, toward the 3′ end of the mRNA [44,45]. A mutation in mouse *RPL24* (*Bst*) causes a pleiotropic developmental phenotype. The hypomorphic mouse mutant allele impairs *RPL24* mRNA splicing and synthesis of the RPL24 protein. While the homozygous allele is lethal, heterozygotes have a reduced protein synthesis rate and altered rRNA ribosome biogenesis [29], while polysome profiles appear quite normal. In contrast, a double mutant in the two *RPL24* genes of *Saccharomyces cerevisiae* is viable. The yeast mutations reduce the growth rate *in vivo* and the protein synthesis rate *in vitro*, probably because of reduced P-site tRNA binding. The *rpl24* mutant yeast polysome profile shows stalled initiation complexes [4,30]. RPL24 being one of the last proteins to be assembled into the 60S [5], it is thought that other ribosomal proteins can join together successfully without RPL24.

### The *rpl24b* mutation does not reveal translation elongation defects or ribosome biogenesis defects

One might consider that a mutation such as *rpl24b* affecting the large ribosomal subunit may cause a translation elongation defect. Such a defect might slow the movement of ribosomes and therefore raise ribosome numbers per mRNA, and thus raise polysome loading, which would appear as an increase in translation state. Such a defect in the translation elongation cycle would be compounded in an exponential way by the length of the protein coding sequence. We did not observe a consistent global increase in ribosome loading when we monitored the RNA absorbance profile of our *rpl24b* mutant polysome gradients. Of note, we also did not detect evidence for half-mers, polysomes with a 40S subunit stalled on the start codon, as were observed in the yeast *rpl24* mutant [31]. Third, the observed increase in ribosome loading in *rpl24b* was gene specific, and preferentially affected the r-protein mRNAs, which are rather short, rather than long mRNAs.

Many ribosomal proteins play fundamental roles in ribosome assembly [5]. However, we are not inclined to conclude that the *rpl24b* phenotype reflects a direct ribosome biogenesis defect, for the main reason that the fundamental aspects of the *rpl24b* mutant phenotype are mirrored by *eif3h*, a mutation in an initiation factor, which would not affect ribosome assembly directly. Taken together, we did not see clear hallmarks of defects in translation elongation or ribosome assembly in *rpl24b*, although we do not categorically rule out that they might exist.

The *rpl24b* mutant stimulated the expression or the ribosome loading of certain r-protein transcripts (Figures 4 and 5). Between the transcriptional and translational upregulation, a large proportion of r-proteins received some boost in expression in the *rpl24b* mutant. A similar result was seen in *eif3h*[20]. It is conceivable that the mutant plants sense the defect in the translation apparatus, for example defective ribosomes lacking RPL24. The molecular phenotype that we observe suggests the existence of a 'translation machinery surveillance’ response, which then stimulates expression of ribosomal proteins.

It is currently not possible to tease apart which components of the *rpl24b* mutant phenotype are due directly to the loss of the RPL24B protein and which are due to the indirect effects of the mutation on other r-proteins. The problem of indirect effects is perhaps even more acute in vertebrates because ribosomal mutations trigger a p53-dependent nucleolar stress response pathway [3]. Whether ribosomal mutations in plants trigger an analogous response is currently unknown.

### Roles of RPL24B consistent with global measurements of translation

Based on transient gene expression assays, *Arabidopsis* RPL24 functions during translation reinitiation on the 35S RNA of cauliflower mosaic virus (CaMV) [22]. Moreover, the *rpl24b* mutant was originally identified as *shortvalve1* by virtue of its short valves, which are the walls of the seed pod [26]. The RPL24 protein boosts the translation reinitiation of two uORF-containing auxin response transcription factor (*ARF*) mRNAs, *ETTIN* (*ETT*) and *MONOPTEROS* (*MP*) [26], in conjunction with eIF3h [25]. Based on this information, we hypothesized that eIF3h and RPL24 may be involved in similar (re)initiation events or may coregulate the expression of uORF-containing or other mRNAs. The data obtained from the global analysis of ribosome loading only partly confirm this hypothesis. Indeed, several dozen mRNAs depended on both eIF3h and RPL24B for regular ribosome loading (Figure 2A). Surprisingly, however, these mRNAs were not highly enriched in uORFs. Vice versa, mRNAs with uORFs that have clearly reduced ribosome loading in *eif3h*, tend to have a rather mild translation defect in *rpl24b* (Additional files 1 and 6). This result shows a certain discrepancy between translation as measured using gene expression assays and translation as measured using ribosome occupancy. The result is actually reminiscent of earlier observations. For example, the *AtbZIP11* mRNA, which is clearly eIF3h dependent and RPL24B dependent in the gene expression assay [20,21,24,25], has a relatively modest and statistically insignificant ribosome occupancy defect when measured by microarray [20]. One probable cause is this; when the ribosome loading is reduced by twofold from six to three ribosomes per mRNA, most mRNA molecules will still be in a 'polysomal’ state. One might further consider that the respective uORF-containing mRNAs remain ribosome loaded in the *rpl24b* mutants because ribosomes persist on their uORF clusters. We can also speculate that mutant *rpl24b* ribosomes might reinitiate translation imperfectly; they may translate portions of the major ORF in an unproductive way that does not lead to a full protein. For comparison, other mutations in the translation apparatus affect the fidelity of start codon recognition [46,47]; aberrant initiation at non-AUG start codons or non-canonical reading frames could potentially be detected using the ribosome footprinting technique [48].

As we compare translation between *rpl24b* and *eif3h*, we should comment on the fact that the two experiments were performed under different light regimes. The *rpl24b* experiment was performed under a 16 h light/8 h dark cycle and the plants were harvested approximately 4 h into the light period, while the other experiments were carried out in continuous light. We do not think that the intermittent dark treatments contributed in a major way to the differences in translation. By examining the mRNA levels of characteristic clock gene transcripts we determined that the continuous-light-grown plants and the *rpl24b* plants were entrained in a similar way by the circadian clock. In addition, when dark-treated plants are reilluminated, the ribosome loading for the majority of mRNAs recovers within 10 minutes [8]. Even when fully etiolated seedlings are exposed to light, the major adjustment in polysome loading occurs within 4 h [10].

### The translational regulon of ribosomal proteins

This work suggests the presence of several translational regulons, defined as groups of mRNAs that are subject to joint translational control across a variety of conditions. We newly noticed that a subset of seed storage protein mRNAs are jointly stimulated in their translation in the *pab2 pab8* mutant. Since this group has not shown coregulation under other conditions, it does not qualify as a regulon as yet. However, r-protein mRNAs clearly form a regulon. Among the r-proteins, the organellar encoded ones are clearly set apart from the nuclear encoded ones. Given their correlated behavior in *rpl24b* and *eif3h*, the organellar r-protein mRNAs appear to be part of a distinct regulon of mRNAs. The r-protein regulon holds steady under other experimental conditions, given that r-protein mRNAs are among the most sensitive to hypoxia and other stresses [6,7]. It is possible that certain r-proteins are excluded from the regulon. However, aside from the 'incomplete’ r-protein mRNAs, it is difficult to identify specific examples. While certain r-proteins seemed to be stimulated less than others by the *rpl24b* and *eif3h* mutations, this could often be attributed to the fact that their translation state was already very high in the wild-type (Figure 5A,B). Thus, it seems plausible that a large fraction of nuclear encoded r-protein mRNAs are subject to coordinated translational regulation. Interestingly, while the response of the r-proteins to mutation is fairly uniform across the entire group, individual r-protein mRNAs vary greatly in their ribosome occupancy (Figure 5B). This result has implications for ascribing functions to individual r-protein paralogs in the context of the hypothesis that the cell contains different ribosomes with specialized functions. When two mutations in sister paralogs display different phenotypes, even though the patterns of transcript abundance of their mRNAs are identical, such differences in phenotype may be due to different rates of translation.

Although the r-protein regulon is functionally the best-characterized plant translational regulon, questions regarding the RNA sequence features responsible for its regulation remain to be addressed in future work. In general, it is unknown what causes the differences in ribosome occupancy among the paralogs. Given that their r-protein coding sequences are highly similar, one must consider whether ribosome occupancy is governed by sequences in the more variable 5′ and 3′ UTRs.

## Conclusions

A mutation affecting a large ribosomal subunit protein, RPL24B, stimulates ribosome loading in a similar way as a mutation in the eukaryotic initiation factor subunit, eIF3h, whereas a mutation in poly(A)-binding protein has a distinct, milder, spectrum of effects. The most striking translational effects are centered on the ribosomal protein mRNAs, suggesting that a large subset of these mRNAs forms a regulon of translational control.

## Methods

### Plant material

*A. thaliana* ecotypes for wild-type plants and alleles and ecotypes for mutants were described previously [21,25,26,34]. Wild-type plants, and the *pab2 pab8* double mutant (alleles SALK_026293 of *PAB2* (At4g34110) and SALK_022160 of *PAB8* (At1g49760)) are in the Columbia-0 (Col-0) ecotype [34]. The *eif3h-1* mutant allele (At1g10840) [21] and *rpl24b* (*stv1-1*) mutant allele (At3g53020) [26] are in the Wassilewskija (Ws) ecotype. The *rpl24b* mutant has a deletion covering several genes neighboring *RPL24B*, and also carries a transgene expressing the small myb protein, CAPRICE at the *RPL24B* locus [26]. Compared to the deletion of *RPL24B*, these additional mutations are likely to have at most small effects, if any, on translation. The *pab2 pab8* mutant is slightly dwarfed in its growth [34]. The mRNA signals were reduced more than 30-fold for *PAB2* and 10-fold for *PAB8* (Additional file 1), consistent with loss of function. The mutation in *eif3h-1* causes a C-terminal truncation of the protein [21]. A T-DNA insertion for *eIF3k* (At4g33250) was recovered from the GABI-KAT collection [49]. The mutant has an insertion in the sixth and last intron and produces a 3′ truncated transcript, but no full length transcript was detected (data not shown). The *eif3k* mutant seedlings lacked any obvious phenotype at the seedling and adult stages.

The *rpl24b* mutant allele *stv1-1* were grown on agar plates containing full strength Murashige and Skoog salts, pH 5.7, and 1% sucrose for 10 to 12 days in a 16 h light/8 h dark cycle at 22°C alongside the corresponding wild-type plants. They were harvested 6 h into the light period. The *pab2 pab8* mutant, the *eif3k* mutant and the *eif3h-1* mutant were grown in continuous light. There were three biological replicates for *rpl24b* and four for *pab2 pab8*.

### RNA extraction and fractionation

The translation efficiency is given by the number of protein molecules synthesized per mRNA molecule per unit time [50]. If one assumes that the rate of elongation, estimated at 5.6 codons/s [48], is approximately uniform across all mRNAs, then a measure of the translational efficiency of an mRNA can be estimated from the ribosome density, defined as the number of ribosomes per length of mRNA, or the ribosome occupancy, defined as the proportion of mRNA molecules found in the polyribosomes.

The entire procedure closely followed that given in [20]. Aerial tissues were homogenized and subjected to sucrose-gradient fractionation to generate two fractions of mRNAs: the non-polysomal fraction (free and monosomal, NP) and the polysomal fraction (PL) (Additional file 7: Figure S5). Samples for total transcripts (TC) were also collected alongside. Following manufacturer’s protocols, PL, NP and T RNA fractions were converted to cDNA and hybridized to GeneChip® Arabidopsis ATH1 Genome Arrays, Affymetrix, Santa Clara, CA which contain more than 22,500 probe sets representing approximately 24,000 genes.

If a given experimental treatment causes a global reduction in polysome loading, the global shift becomes masked during the standardized experimental procedure. The global shift is measured using polysome UV-absorbance profiles and RNA abundance measurements [9]. In our hands, neither the *rpl24b* mutant nor the *pab2 pab8* mutant showed any reproducible global shift in polysome loading (not shown). Therefore, no global adjustment was performed. Because we cannot rule out that small global shifts in polysome loading did occur in the mutants, our data only speak to gene-specific changes.

### Microarray data analysis

Raw signal intensities for each probe set were extracted from CEL files (Affymetrix proprietary data format) using the open source statistical tool R/Bioconductor [51], and the affy package [52]. We compared three background correction and normalization methods, MAS5, Robust Multi-array Average, and gcRMA. We settled on the Robust Multi-array Average algorithm because it minimized the variance in TL among the biological replicates (Additional file 8: Figure S6A,B) and was more successful in identifying differentially translated genes as judged from volcano plots (Additional file 8: Figure S6C-F). Therefore, the two replicates of raw data on *eif3h-1*[20] were re-extracted using RMA. Translation states (TL) were calculated as the ratio between PL and NP as TL = (PL)/(NP) for mutant and wild-type samples and were displayed after log_2_ transformation. The ratio in TL between the respective mutant and wild-type is given as a log-difference by Δlog_2_ TL = log_2_ (TL_mut_/TL_WT_) = log_2_ TL_mut_ - log_2_ TL_WT_. Differences in the total transcript level (TC) are also given as log_2_ ratios, as usual.

### Filtering

The complete set of genes (n = 22,746) represented on the GeneChip® Arabidopsis ATH1 Genome Array microarray ('All’) was prefiltered based on one of two simple gene expression criteria. First, Present (P), Marginal (M), or Absent (A) calls were recorded using the *mas5calls* function of the Bioconductor *MAS5* algorithm. For the 'P/M’ set, only those genes were included that had 'P’ and/or 'M’ calls in at least 50% of the arrays of each dataset (n = 8 to 16). Second, for the 'Stable’ set, we selected only genes that had a low variance in their translation state across a panel of 12 wild-type samples (see below). Prefiltering yielded four gene sets, that is, 'All’ , 'P/M’ , 'Stable’ and 'Stable and P/M’. While some differentially translated genes may be lost during prefiltering, the reduced size of the prefiltered data sets increases the statistical power.

### Identification of differentially translated genes (DTGs)

Genes that are differentially translated (DTGs) between mutant and wild-type plants were identified in two ways. First, we took all four prefiltered sets defined above and applied limma with a cut-off against false-positive discovery of 0.05 (FDR_BH_ 5%) to adjust aggressively for errors due to multiple-hypothesis testing [53,54] and at least a twofold difference in TL. Any gene identified by any one of the four prefilters and passing limma/FDR was considered a DTG. An alternate method ('*ad hoc’* filtering, [20]) simply examines the replicates for their fold change between mutant and wild-type, as well as the coefficient of variation (CV) thereof. With the *ad hoc* filter, genes were selected based on any one of three criteria; (i) a fold change of more than twofold (log_2_ ≥1.0) in all replicates; (ii) any fold change (as log_2_) with a coefficient of variation of less than 50%; (iii) any fold change (as log_2_) with a SD of less than 0.5. The *ad hoc* method captures most genes identified by limma/FDR, but also preserves reproducibly unchanged genes as well as genes whose up or down trend is clear but too noisy to withstand more rigorous FDR filtering (Additional file 9: Figure S7). To define genes with differential transcript levels (Δlog_2_ TC) only the limma/FDR method was used.

### Clustering and higher level analyses

Higher level analyses of DTGs utilized fuzzy *k*-means clustering as described previously [8,55,56]. Briefly, to identify coregulated genes, clusters of mRNAs were resolved by fuzzy *k*-means clustering with the fanny function of the R cluster package [57] using the following settings: distance measure = Euclidean correlation, membership exponent = 1.1, maximal number of iterations = 5,000, and number of clusters determined by trial and error. Clusters of genes and values of fold changes (Δlog_2_ TL) of mRNAs were visualized as heatmaps with the graphical interface programs MEV [58] and Genesis [59].

Groups of genes identified by clustering or other means were evaluated for enrichment of functional annotations using the Genome Ontology tools GOHyperGAll function [60] and the DAVID (Database for Annotation, Visualization and Integrated Discovery) database [61]. Moreover, fold-change values of translation states (Δlog_2_ TL) of the whole genome (n = 22,746) were plotted onto *Arabidopsis* biochemical pathways and analyzed for functional bias of gene expression using MapMan v3.5.1R2 [62]. Ribosomal protein annotations were identified from [42,43].

### Definition of mRNAs with stable translation states

Translation states (TL) of mRNAs from wild-type seedlings were tabulated for 12 biological replicates from 4 separate experiments. These samples were the wild-type control samples from experiments performed with *eif3h-1* (2 replicates), *rpl24b* (3 replicates), *pab2 pab8* (4 replicates) and *eif3k* (3 replicates). Data from 'All’ probesets were retained without any prefiltering. The standard deviations were calculated across all 12 datasets. The top 20% of genes with the highest standard deviations were classified as genes with highly variable translation state; while the remainder was classified as 'Stable’. Among the latter, genes devoid of any 'MAS5 Absent’ calls were classified as 'Stable and P/M’.

### Accession numbers for the data

New array data were submitted to the Gene Expression Omnibus database [63] under the following accession numbers: *pab2 pab8*: GEO - GSE51480; *rpl24b*: GEO - GSE51474; *eif3k*: GEO - GSE28224. Previously published data are found here: *eif3h* mutant [20]: GEO - GSE6024 (TL) and GEO - GSE6025 (TC).

## Competing interests

The authors declare that they have no competing interests.

## Authors’ contributions

BST performed bioinformatic and statistical analyses. BHK performed experimental work and contributed bioinformatic analyses. DRG and BR contributed reagents and advice. AGvA and DRG conceived of the study. AGvA guided all aspects of the project. BST and AGvA wrote the article. All authors read and approved the final manuscript.

## Supplementary Material

Additional file 1 **Identification of differentially translated genes from *rpl24b *** **and *eif3h* and *pab2 pab8* mutant seedlings using four different prestatistical filtering methods.** See Methods for definition of the datasets 'All’, 'P/M’, 'Stable’, and 'Stable and P/M’. Click here for file

Additional file 2: Figure S1 The translational defects in the *rpl24b* mutant are not correlated with changes in total transcript levels, similar to *eif3h*[20]. Scatterplots show comparisons of global changes in total transcript (y-axes) against the respective changes in translation state (TL) (Δlog_2_ TL = Δlog_2_ polysomal fraction (PL)/non-polysomal fraction (NP)) for *rpl24b* and *eif3h* mutants compared to wild-type samples. All *Arabidopsis thaliana* genes on GeneChip® Arabidopsis ATH1 Genome Array (n = 22,746) were analyzed and Pearson correlation coefficients (R^2^) are indicated. Dashed lines represent twofold changes for each comparison between the respective mutant sample and wild-type in upward (red) or downward (blue) direction, respectively. Click here for file

Additional file 3: Figure S2 Differentially translated genes in *rpl24b* and *eif3h* mutant seedlings are enriched for r-protein mRNAs. The analysis follows that in Figure 2, except that differentially translated genes (DTGs) were identified by an *ad hoc* method (see Methods) rather than limma/false discovery rate (FDR). In addition, we required at least a 1.5-fold change (log_2_ = 0.59) in translation state (TL) in *rpl24b* or *eif3h*, and excluded genes that did not have P(resent) or M(arginal) calls in at least 50% of arrays. **(A)** Scatterplot showing the translational codependence of sets of mRNAs on *RPL24B* and/or *eIF3h*. Pearson correlation coefficients (R^2^) are indicated. Dashed lines represent 1.5-fold changes. Black dots indicate mRNAs that pass the filter in only one mutant but not the other. Red and blue dots indicate mRNAs that pass in both mutants. **(B)** Venn diagrams showing overlap for genes undertranslated (top) or overtranslated (bottom) in the *eif3h* and *rpl24b* mutant. **(C)** Heatmaps of fuzzy k-means clustering of 1,985 differentially translated genes (|Δlog_2_ TL| ≥0.59). The heatmaps display median Δlog_2_ TL values for each of seven clusters of mRNAs that had similar translation defects in each mutant over wild-type. The number of genes in each cluster is indicated. Translation defects are generally correlated for subsets of *rpl24b* and *eif3h* mistranslated mRNAs (the first five columns). The translation state data observed in the *pab2 pab8* mutant and in the *eif3k* mutant are shown for comparison; these were added after clustering had been performed. From left to right columns, data are for three (*rpl24b*), two (*eif3h*), four (*pab2 pab8*), and two (*eif3k*) experimental replicates. The heatmap color panel indicates undertranslation (blue), overtranslation (yellow) and no change (white) in mRNA translation. **(D)** Each gene cluster was examined for enrichment of functional categories. Enrichment *P* values were calculated by the GOHyperGAll function. GO terms: BP, biological process; CC, cellular component; MF, molecular function. Click here for file

Additional file 4: Figure S3 Ribosome loading of mRNA for organellar r-proteins in *rpl24b* and *eif3h* mutant plants. The heatmap shows the ribosomal occupancy defects in *rpl24b* and *eif3h* mutants of all paralogous mRNAs for plastidic and mitochondrial r-proteins that are represented on the ATH1 microarray. Those r-proteins that are encoded in the plastids and mitochondria are highlighted with asterisks, while the remainder are nuclear encoded. Yellow and blue represent that ribosome occupancy in the mutant is stimulated and inhibited, respectively. Gray cells indicate that the gene does not exist or did not yield data. Click here for file

Additional file 5 **Translation states (TL) of ribosomal proteins in the *rpl24b *** **mutant and the *eif3h* mutant displayed in Figure **5 and Additional file 4: Figure S3. Click here for file

Additional file 6: Figure S4 Upstream open reading frames (uORFs) and longer coding sequences contribute to poor translatability of mRNAs in *rpl24b* and *eif3h* mutants. The contribution of the length of main open reading frame (ORF or CDS) and the presence of uORFs to the translation state (TL) of mRNAs. mRNAs were classified into bins according to differences in translation state (Δlog_2_ TL) between *rpl24b* and wild-type **(A)** or *eif3h* and wild-type **(B)**. Each bin was evaluated for the percentage of genes falling into three classes (i) genes harboring uORFs; (ii) genes lacking uORFs but having a long (>1,086 nt) ORF; (iii) genes lacking uORFs but with a short (<1,086 nt) ORF. The number of genes in each class is indicated. The 2 × 2 contingency tables were prepared from the 'no change’ bin (> - 0.2 Δlog_2_ TL <0.2), and each of the other bins. Fisher’s exact test (or χ^2^ test with Yates’ correction for the larger classes) was carried out using these tables to evaluate the extent of deviation of each bin from the 'no change’ bin. Significant (>0.0001 *P* <0.05) and highly significant (*P* <0.0001) deviations are shown with single and double asterisks, respectively. (C) mRNAs that depend specifically on *eIF3h* are strongly enriched for uORFs, while mRNAs that depend on both *eIF3h* and *RPL24B* are not. Click here for file

Additional file 7: Figure S5 UV absorbance profiles (254 nm) were collected during gradient fractionation. **(A)***rpl24b* mutant and corresponding wild-type. **(B)***pab2 pab8* mutant and corresponding wild-type. Labels indicate the position of the 40S (peak), 60S (shoulder), and 80S (peak) ribosomes as well as polysomes with two and four ribosomes. Click here for file

Additional file 8: Figure S6 The Robust Multi-array Average algorithm minimizes standard deviations in microarray data of mRNA translation state (TL; ribosome occupancy). **(A)** Averages of translation states (log_2_ TL) were calculated for four replicates of wild-type reference samples drawn from the *pab2 pab8* mutant experiment. **(B)** Genes were binned according to the standard deviations (SD) of their translation state. All *Arabidopsis* genes (n = 22,746) represented on the GeneChip® Arabidopsis ATH1 Genome Array were analyzed. Note that the fraction of genes with SD >0.4 is minimized when using RMA. Reference samples (mock treatment) taken from hypoxia [6] and Turnip Mosaic Virus (TuMV) infection [12] datasets also showed similar distributions with RMA consistently giving lower variability (SD <0.5) of log_2_ TL values between replicates (not shown). **(C-F)** Comparison of the number of differentially translated genes (DTGs) between RMA-normalized and gcRMA-normalized data. Changes in translation state (Δlog_2_ TL) of RMA-normalized (C,D) and gcRMA-normalized (E,F) data for the *rpl24b* and *eif3h* datasets. Values on the x-axes show the fold changes in translation state in the respective mutant over wild-type samples. Y-axes show statistical significance of these changes according to limma/false discovery rate (FDR) adjusted *P* values, log transformed ('volcano plot’). Horizontal dashed lines mark the FDR <0.05 (-log value = 1.3) cut-off. The vertical dashed lines delineate 1.5-fold changes in upward (red) or downward (blue) directions. All *Arabidopsis* genes (n = 22,746) represented on GeneChip® Arabidopsis ATH1 Genome Array were analyzed. In (F) the legend explains the color coding used to illustrate different levels of significance. Click here for file

Additional file 9: Figure S7 Comparison between two methods for identifying differentially translated genes (DTGs), the false discovery rate (FDR)-validated method, and the *ad hoc* method. The figure shows all 18,757 genes that pass the *ad hoc* filter. Of these, the limma/FDR method with a twofold cut-off identified the genes marked in red and blue. When used with a twofold cut-off criterion, both methods identify very similar sets of mRNAs, but the *ad hoc* method also identifies those labeled green and orange. In contrast, when used with a 1.5-fold cut-off criterion (stippled lines), the *ad hoc* method yields a large number of additional genes, lying above and below the stippled lines, that have modest standard deviations (<0.5) but that are not selected by the limma/FDR method unless one relaxes the false-discovery criterion from 0.05 to as far as 0.4. s, significant by limma with an FDR threshold of <0.05; ns, FDR >0.05. Click here for file

## References

[B1] Horiguchi G Van Lijsebettens M Candela H Micol JL Tsukaya H Ribosomes and translation in plant developmental control Plant Sci 2012 11 24 34 22682562 10.1016/j.plantsci.2012.04.008

[B2] McCann KL Baserga SJ Genetics. Mysterious ribosomopathies Science 2013 11 849 850 10.1126/science.1244156 23970686 PMC3893057

[B3] Terzian T Box N Genetics of ribosomal proteins: “curiouser and curiouser” PLoS Genet 2013 11 e1003300 10.1371/journal.pgen.1003300 23382707 PMC3561088

[B4] Horos R Ijspeert H Pospisilova D Sendtner R Andrieu-Soler C Taskesen E Nieradka A Cmejla R Sendtner M Touw IP von Lindern M Ribosomal deficiencies in Diamond-Blackfan anemia impair translation of transcripts essential for differentiation of murine and human erythroblasts Blood 2012 11 262 272 10.1182/blood-2011-06-358200 22058113

[B5] Kondrashov N Pusic A Stumpf CR Shimizu K Hsieh AC Xue S Ishijima J Shiroishi T Barna M Ribosome-mediated specificity in Hox mRNA translation and vertebrate tissue patterning Cell 2011 11 383 397 10.1016/j.cell.2011.03.028 21529712 PMC4445650

[B6] Branco-Price C Kaiser KA Jang CJ Larive CK Bailey-Serres J Selective mRNA translation coordinates energetic and metabolic adjustments to cellular oxygen deprivation and reoxygenation in *Arabidopsis thaliana* Plant J 2008 11 743 755 10.1111/j.1365-313X.2008.03642.x 18665916

[B7] Branco-Price C Kawaguchi R Ferreira RB Bailey-Serres J Genome-wide analysis of transcript abundance and translation in *Arabidopsis* seedlings subjected to oxygen deprivation Ann Bot 2005 11 647 660 10.1093/aob/mci217 16081496 PMC4247032

[B8] Juntawong P Bailey-Serres J Dynamic light regulation of translation status in *Arabidopsis thaliana* Frontiers Plant Sci 2012 11 66 10.3389/fpls.2012.00066 PMC3355768 22645595

[B9] Kawaguchi R Girke T Bray EA Bailey-Serres J Differential mRNA translation contributes to gene regulation under non-stress and dehydration stress conditions in *Arabidopsis thaliana* Plant J 2004 11 823 839 10.1111/j.1365-313X.2004.02090.x 15144383

[B10] Liu MJ Wu SH Chen HM Widespread translational control contributes to the regulation of *Arabidopsis* photomorphogenesis Mol Syst Biol 2012 11 566 22252389 10.1038/msb.2011.97 PMC3296358

[B11] Matsuura H Ishibashi Y Shinmyo A Kanaya S Kato K Genome-wide analyses of early translational responses to elevated temperature and high salinity in *Arabidopsis thaliana* Plant Cell Physiol 2010 11 448 462 10.1093/pcp/pcq010 20089509

[B12] Moeller JR Moscou MJ Bancroft T Skadsen RW Wise RP Whitham SA Differential accumulation of host mRNAs on polyribosomes during obligate pathogen-plant interactions Molecular Biosyst 2012 11 2153 2165 10.1039/c2mb25014d 22660698

[B13] Nicolai M Roncato MA Canoy AS Rouquie D Sarda X Freyssinet G Robaglia C Large-scale analysis of mRNA translation states during sucrose starvation in arabidopsis cells identifies cell proliferation and chromatin structure as targets of translational control Plant Physiol 2006 11 663 673 10.1104/pp.106.079418 16632591 PMC1475480

[B14] Sormani R Delannoy E Lageix S Bitton F Lanet E Saez-Vasquez J Deragon JM Renou JP Robaglia C Sublethal cadmium intoxication in *Arabidopsis thaliana* impacts translation at multiple levels Plant Cell Physiol 2011 11 436 447 10.1093/pcp/pcr001 21252299

[B15] Ueda K Matsuura H Yamaguchi M Demura T Kato K Genome-wide analyses of changes in translation state caused by elevated temperature in *Oryza sativa* Plant Cell physiol 2012 11 1481 1491 10.1093/pcp/pcs092 22722767

[B16] Yanguez E Castro-Sanz AB Fernandez-Bautista N Oliveros JC Castellano MM Analysis of genome-wide changes in the translatome of *Arabidopsis* seedlings subjected to heat stress PLoS One 2013 11 e71425 10.1371/journal.pone.0071425 23977042 PMC3747205

[B17] Roy B von Arnim AG Chang C, Torii K Translational regulation of cytoplasmic mRNAs The Arabidopsis Book 2013 11 Rockville, MD: American Society of Plant Biologists e0165 23908601 10.1199/tab.0165 PMC3727577

[B18] Mayberry LK Allen ML Dennis MD Browning KS Evidence for variation in the optimal translation initiation complex: plant eIF4B, eIF4F, and eIF(iso)4 F differentially promote translation of mRNAs Plant Physiol 2009 11 1844 1854 10.1104/pp.109.138438 19493973 PMC2719132

[B19] Mayberry LK Allen ML Nitka KR Campbell L Murphy PA Browning KS Plant cap-binding complexes eukaryotic initiation factors eIF4F and eIFiso4F: molecular specificity of subunit binding J Biol Chem 2011 11 42566 42574 10.1074/jbc.M111.280099 21965660 PMC3234931

[B20] Kim BH Cai X Vaughn JN von Arnim AG On the functions of the h subunit of eukaryotic initiation factor 3 in late stages of translation initiation Genome Biol 2007 11 R60 10.1186/gb-2007-8-4-r60 17439654 PMC1896003

[B21] Kim TH Kim BH Yahalom A Chamovitz DA von Arnim AG Translational regulation via 5′ mRNA leader sequences revealed by mutational analysis of the *Arabidopsis* translation initiation factor subunit eIF3h Plant Cell 2004 11 3341 3356 10.1105/tpc.104.026880 15548739 PMC535877

[B22] Park HS Himmelbach A Browning KS Hohn T Ryabova LA A plant viral “reinitiation” factor interacts with the host translational machinery Cell 2001 11 723 733 10.1016/S0092-8674(01)00487-1 11572778

[B23] Schepetilnikov M Dimitrova M Mancera-Martinez E Geldreich A Keller M Ryabova LA TOR and S6K1 promote translation reinitiation of uORF-containing mRNAs via phosphorylation of eIF3h EMBO J 2013 11 1087 1102 10.1038/emboj.2013.61 23524850 PMC3630359

[B24] Roy B Vaughn JN Kim BH Zhou F Gilchrist MA von Arnim AG The h subunit of eIF3 promotes reinitiation competence during translation of mRNAs harboring upstream open reading frames RNA 2010 11 748 761 10.1261/rna.2056010 20179149 PMC2844622

[B25] Zhou F Roy B von Arnim AG Translation reinitiation and development are compromised in similar ways by mutations in translation initiation factor eIF3h and the ribosomal protein RPL24 BMC Plant Biol 2010 11 193 10.1186/1471-2229-10-193 20799971 PMC3020687

[B26] Nishimura T Wada T Yamamoto KT Okada K The *Arabidopsis* STV1 protein, responsible for translation reinitiation, is required for auxin-mediated gynoecium patterning Plant Cell 2005 11 2940 2953 10.1105/tpc.105.036533 16227452 PMC1276021

[B27] Rosado A Raikhel NV Application of the gene dosage balance hypothesis to auxin-related ribosomal mutants in *Arabidopsis* Plant Signal Behav 2010 11 450 452 10.4161/psb.5.4.11341 20383066 PMC2958597

[B28] Pendle AF Clark GP Boon R Lewandowska D Lam YW Andersen J Mann M Lamond AI Brown JW Shaw PJ Proteomic analysis of the *Arabidopsis* nucleolus suggests novel nucleolar functions Mol Biol Cell 2005 11 260 269 15496452 10.1091/mbc.E04-09-0791 PMC539170

[B29] Oliver ER Saunders TL Tarle SA Glaser T Ribosomal protein L24 defect in belly spot and tail (Bst), a mouse Minute Development 2004 11 3907 3920 10.1242/dev.01268 15289434 PMC2262800

[B30] Dresios J Derkatch IL Liebman SW Synetos D Yeast ribosomal protein L24 affects the kinetics of protein synthesis and ribosomal protein L39 improves translational accuracy, while mutants lacking both remain viable Biochemistry 2000 11 7236 7244 10.1021/bi9925266 10852723

[B31] Baronas-Lowell DM Warner JR Ribosomal protein L30 is dispensable in the yeast Saccharomyces cerevisiae Mol Cell Biol 1990 11 5235 5243 2204809 10.1128/mcb.10.10.5235-5243.1990 PMC361207

[B32] Kruiswijk T Planta RJ Krop JM The course of the assembly of ribosomal subunits in yeast Biochim Biophys Acta 1978 11 378 389 10.1016/0005-2787(78)90204-6 626744

[B33] Belostotsky DA Unexpected complexity of poly(A)-binding protein gene families in flowering plants: three conserved lineages that are at least 200 million years old and possible auto- and cross-regulation Genetics 2003 11 311 319 12586718 10.1093/genetics/163.1.311 PMC1462424

[B34] Dufresne PJ Ubalijoro E Fortin MG Laliberte JF *Arabidopsis thaliana* class II poly(A)-binding proteins are required for efficient multiplication of turnip mosaic virus J Gen Virol 2008 11 2339 2348 10.1099/vir.0.2008/002139-0 18753244

[B35] Palanivelu R Belostotsky DA Meagher RB Conserved expression of *Arabidopsis thaliana* poly(A) binding protein 2 (PAB2) in distinct vegetative and reproductive tissues Plant J 2000 11 199 210 10.1046/j.1365-313x.2000.00720.x 10849338

[B36] Iwakawa HO Tajima Y Taniguchi T Kaido M Mise K Tomari Y Taniguchi H Okuno T Poly(A)-binding protein facilitates translation of an uncapped/nonpolyadenylated viral RNA by binding to the 3′ untranslated region J Virol 2012 11 7836 7849 10.1128/JVI.00538-12 22593149 PMC3421650

[B37] Cheng S Gallie DR Competitive and noncompetitive binding of eIF4B, eIF4A, and the poly(A) binding protein to wheat translation initiation factor eIFiso4G Biochemistry 2010 11 8251 8265 10.1021/bi1008529 20795652

[B38] Kahvejian A Svitkin YV Sukarieh R M’Boutchou MN Sonenberg N Mammalian poly(A)-binding protein is a eukaryotic translation initiation factor, which acts via multiple mechanisms Genes Dev 2005 11 104 113 10.1101/gad.1262905 15630022 PMC540229

[B39] Sachs AB Varani G Eukaryotic translation initiation: there are (at least) two sides to every story Nat Struct Biol 2000 11 356 361 10.1038/75120 10802729

[B40] Muench DG Zhang C Dahodwala M Control of cytoplasmic translation in plants Wiley Interdiscip Rev RNA 2012 11 178 194 10.1002/wrna.1104 22215505

[B41] Lange H Sement FM Canaday J Gagliardi D Polyadenylation-assisted RNA degradation processes in plants Trends Plant Sci 2009 11 497 504 10.1016/j.tplants.2009.06.007 19716749

[B42] Barakat A Szick-Miranda K Chang IF Guyot R Blanc G Cooke R Delseny M Bailey-Serres J The organization of cytoplasmic ribosomal protein genes in the *Arabidopsis* genome Plant Physiol 2001 11 398 415 10.1104/pp.010265 11598216 PMC125077

[B43] Sormani R Masclaux-Daubresse C Daniel-Vedele F Chardon F Transcriptional regulation of ribosome components are determined by stress according to cellular compartments in *Arabidopsis thaliana* PLoS One 2011 11 e28070 10.1371/journal.pone.0028070 22164228 PMC3229498

[B44] Armache JP Jarasch A Anger AM Villa E Becker T Bhushan S Jossinet F Habeck M Dindar G Franckenberg S Marquez V Mielke T Thomm M Berninghausen O Beatrix B Söding J Westhof E Wilson DN Beckmann R Localization of eukaryote-specific ribosomal proteins in a 5.5-A cryo-EM map of the 80S eukaryotic ribosome Proc Natl Acad Sci U S A 2010 11 19754 19759 10.1073/pnas.1010005107 20974910 PMC2993421

[B45] Klinge S Voigts-Hoffmann F Leibundgut M Ban N Atomic structures of the eukaryotic ribosome Trends Biochem Sci 2012 11 189 198 10.1016/j.tibs.2012.02.007 22436288

[B46] Hinnebusch AG Molecular mechanism of scanning and start codon selection in eukaryotes Microbiol Mol Biol Rev 2011 11 434 467 10.1128/MMBR.00008-11 21885680 PMC3165540

[B47] Hiraishi H Shin BS Udagawa T Nemoto N Chowdhury W Graham J Cox C Reid M Brown SJ Asano K Interaction between 25S rRNA A loop and eukaryotic translation initiation factor 5B promotes subunit joining and ensures stringent AUG selection Mol Cell Biol 2013 11 3540 3548 10.1128/MCB.00771-13 23836883 PMC3753867

[B48] Ingolia NT Lareau LF Weissman JS Ribosome profiling of mouse embryonic stem cells reveals the complexity and dynamics of mammalian proteomes Cell 2011 11 789 802 10.1016/j.cell.2011.10.002 22056041 PMC3225288

[B49] Rosso MG Li Y Strizhov N Reiss B Dekker K Weisshaar B An *Arabidopsis thaliana* T-DNA mutagenized population (GABI-Kat) for flanking sequence tag-based reverse genetics Plant Mol Biol 2003 11 247 259 14756321 10.1023/B:PLAN.0000009297.37235.4a

[B50] Mathews MB Sonenberg N Hershey JWB Mathews MB, Sonenberg N, Hershey JWB Origins and principles of translational control Translational Control in Biology and Medicine 2007 Cold Spring Harbor, NY: CSHL Press 1 40

[B51] Saeed AI Sharov V White J Li J Liang W Bhagabati N Braisted J Klapa M Currier T Thiagarajan M Sturn A Snuffin M Rezantsev A Popov D Ryltsov A Kostukovich E Borisovsky I Liu Z Vinsavich A Trush V Quackenbush J TM4: a free, open-source system for microarray data management and analysis Biotechniques 2003 11 374 378 12613259 10.2144/03342mt01

[B52] Wettenhall JM Simpson KM Satterley K Smyth GK affylmGUI: a graphical user interface for linear modeling of single channel microarray data Bioinformatics 2006 11 897 899 10.1093/bioinformatics/btl025 16455752

[B53] Benjamini Y Hochberg Y Controlling the false discovery rate: a practical and powerful approach to multiple testing J Roy Statist Soc B 1995 11 289 300

[B54] Smyth GK Gentleman VR, Carey SD, Irizarry R, Huber W Limma: linear models for microarray data Bioinformatics and Computational Biology Solutions using R and Bioconductor 2005 New York, NY: Springer 397 420

[B55] Gasch AP Eisen MB Exploring the conditional coregulation of yeast gene expression through fuzzy k-means clustering Genome Biol 2002 11 R59 10.1186/gb-2002-3-11-research0059 PMC133443 12429058

[B56] Gath I Unsupervised optimalf fuzzy clustering IEEE Trans Pattern Anal Mach Intell 1989 11 773 780 10.1109/34.192473

[B57] Maechler M Rousseuw P Struyf A Hubert M Hornik K 2012 cluster: cluster analysis basics and extensions. R package version 1.14.3 [http://cran.r-project.org/web/packages/cluster/index.html]

[B58] Dana-Farber Cancer Institute TM4 [http://www.tm4.org/mev/credits/references]

[B59] Sturn A Quackenbush J Trajanoski Z Genesis: cluster analysis of microarray data Bioinformatics 2002 11 207 208 10.1093/bioinformatics/18.1.207 11836235

[B60] Horan K Jang C Bailey-Serres J Mittler R Shelton C Harper JF Zhu JK Cushman JC Gollery M Girke T Annotating genes of known and unknown function by large-scale coexpression analysis Plant Physiol 2008 11 41 57 10.1104/pp.108.117366 18354039 PMC2330292

[B61] da Huang W Sherman BT Lempicki RA Systematic and integrative analysis of large gene lists using DAVID bioinformatics resources Nat Protoc 2009 11 44 57 19131956 10.1038/nprot.2008.211

[B62] Thimm O Blasing O Gibon Y Nagel A Meyer S Krüger P Selbig J Muller LA Rhee SY Stitt M MAPMAN: a user-driven tool to display genomics data sets onto diagrams of metabolic pathways and other biological processes Plant J 2004 11 914 939 10.1111/j.1365-313X.2004.02016.x 14996223

[B63] Barrett T Wilhite SE Ledoux P Evangelista C Kim IF Tomashevsky M Marshall KA Phillippy KH Sherman PM Holko M Yefanov A Lee H Zhang N Robertson CL Serova N Davis S Soboleva A NCBI GEO: archive for functional genomics data sets–update Nucleic Acids Res 2013 11 D991 D995 10.1093/nar/gks1193 23193258 PMC3531084

