# Supplementary figures and images for: The global translation profile in a ribosomal protein mutant resembles that of an eIF3 mutant
Source: BMC Biol. 2013 Dec 30;11:123. doi: 10.1186/1741-7007-11-123 (PMC3901033; doi:10.1186/1741-7007-11-123)

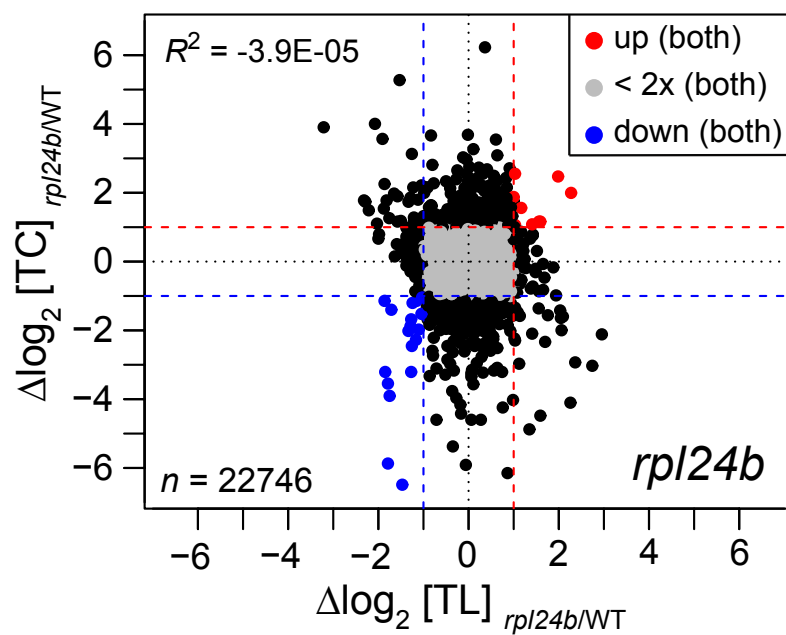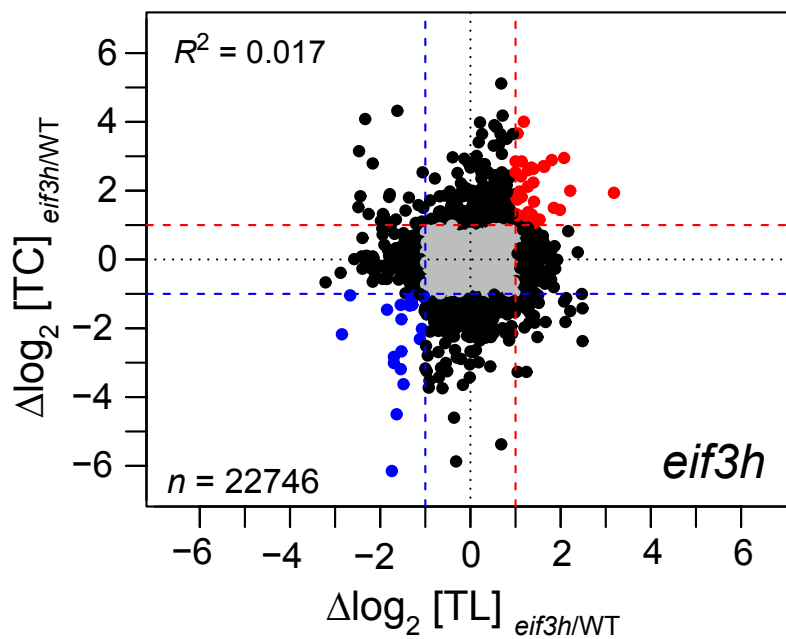

Supplement: Additional file 2: Figure S1 — The translational defects in the rpl24b mutant are not correlated with changes in total transcript levels, similar to eif3h[20]. Scatterplots show comparisons of global changes in total transcript (y-axes) against the respective changes in translation state (TL) (Δlog2 TL = Δlog2 polysomal fraction (PL)/non-polysomal fraction (NP)) for rpl24b and eif3h mutants compared to wild-type samples. All Arabidopsis thaliana genes on GeneChip® Arabidopsis ATH1 Genome Array (n = 22,746) were analyzed and Pearson correlation coefficients (R2) are indicated. Dashed lines represent twofold changes for each comparison between the respective mutant sample and wild-type in upward (red) or downward (blue) direction, respectively. [file 1741-7007-11-123-S2.pdf]

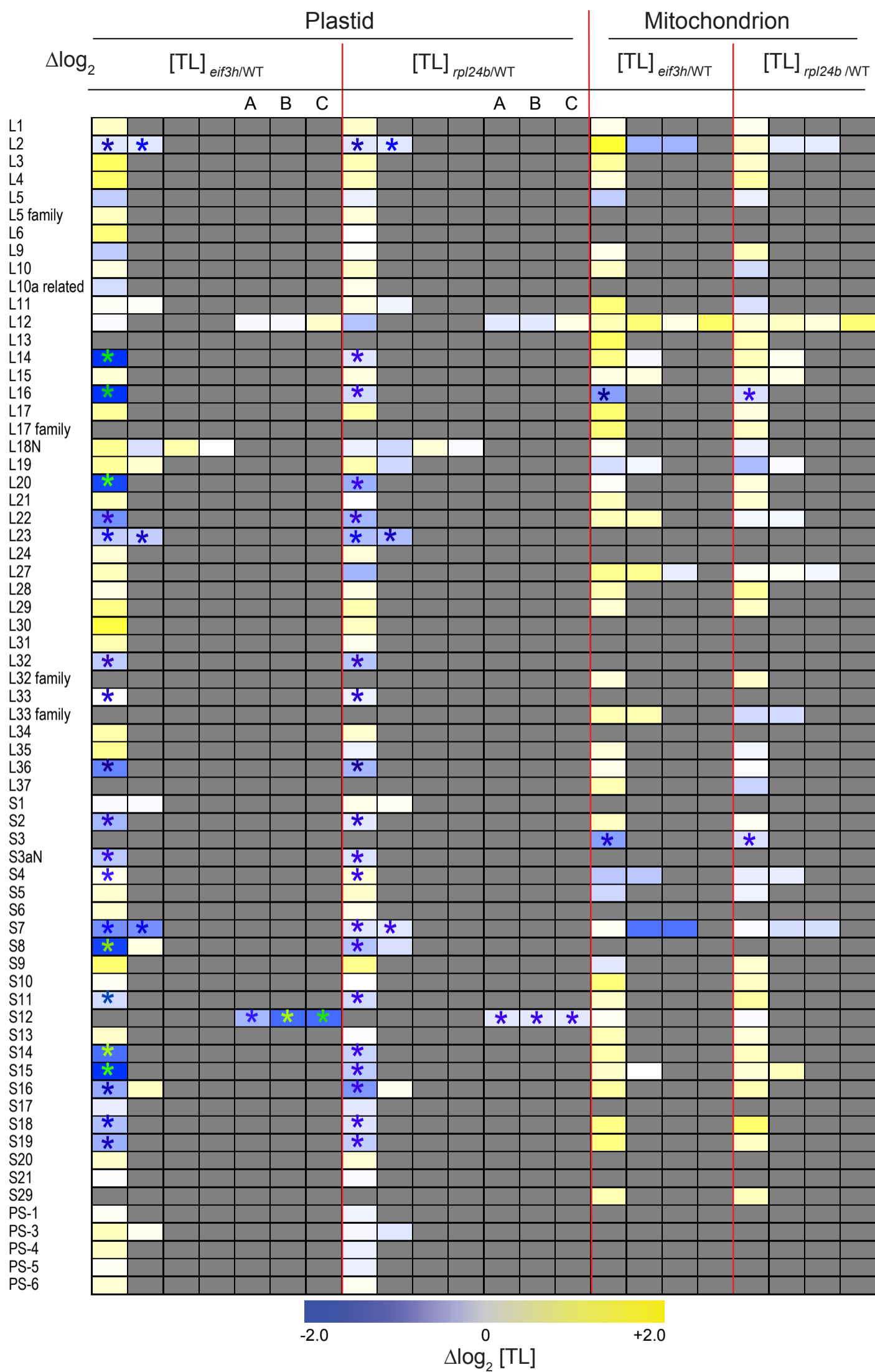

Supplement: Additional file 4: Figure S3 — Ribosome loading of mRNA for organellar r-proteins in rpl24b and eif3h mutant plants. The heatmap shows the ribosomal occupancy defects in rpl24b and eif3h mutants of all paralogous mRNAs for plastidic and mitochondrial r-proteins that are represented on the ATH1 microarray. Those r-proteins that are encoded in the plastids and mitochondria are highlighted with asterisks, while the remainder are nuclear encoded. Yellow and blue represent that ribosome occupancy in the mutant is stimulated and inhibited, respectively. Gray cells indicate that the gene does not exist or did not yield data. [file 1741-7007-11-123-S4.pdf]

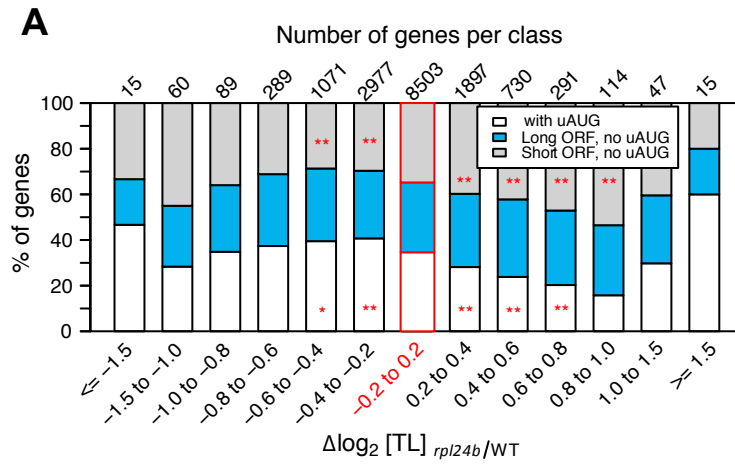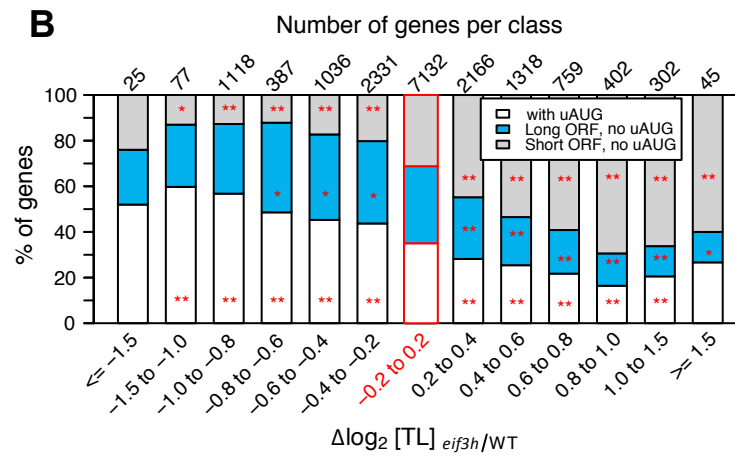

**C**

| $\Delta \text{TL}$ |               | Number of genes |         |           |             |
|--------------------|---------------|-----------------|---------|-----------|-------------|
| <i>eif3h</i>       | <i>rpl24b</i> | Total           | no uORF | with uORF | % with uORF |
| < -1.0             | < -1.0        | 21              | 16      | 5         | 24%         |
| < -1.0             | < -0.6        | 30              | 22      | 8         | 27%         |
| < -1.0             | > -0.6        | 24              | 8       | 16        | 67%         |

Supplement: Additional file 6: Figure S4 — Upstream open reading frames (uORFs) and longer coding sequences contribute to poor translatability of mRNAs in rpl24b and eif3h mutants. The contribution of the length of main open reading frame (ORF or CDS) and the presence of uORFs to the translation state (TL) of mRNAs. mRNAs were classified into bins according to differences in translation state (Δlog2 TL) between rpl24b and wild-type (A) or eif3h and wild-type (B). Each bin was evaluated for the percentage of genes falling into three classes (i) genes harboring uORFs; (ii) genes lacking uORFs but having a long (>1,086 nt) ORF; (iii) genes lacking uORFs but with a short (<1,086 nt) ORF. The number of genes in each class is indicated. The 2 × 2 contingency tables were prepared from the 'no change’ bin (> - 0.2 Δlog2 TL <0.2), and each of the other bins. Fisher’s exact test (or χ2 test with Yates’ correction for the larger classes) was carried out using these tables to evaluate the extent of deviation of each bin from the 'no change’ bin. Significant (>0.0001 P <0.05) and highly significant (P <0.0001) deviations are shown with single and double asterisks, respectively. (C) mRNAs that depend specifically on eIF3h are strongly enriched for uORFs, while mRNAs that depend on both eIF3h and RPL24B are not. [file 1741-7007-11-123-S6.pdf]

**A**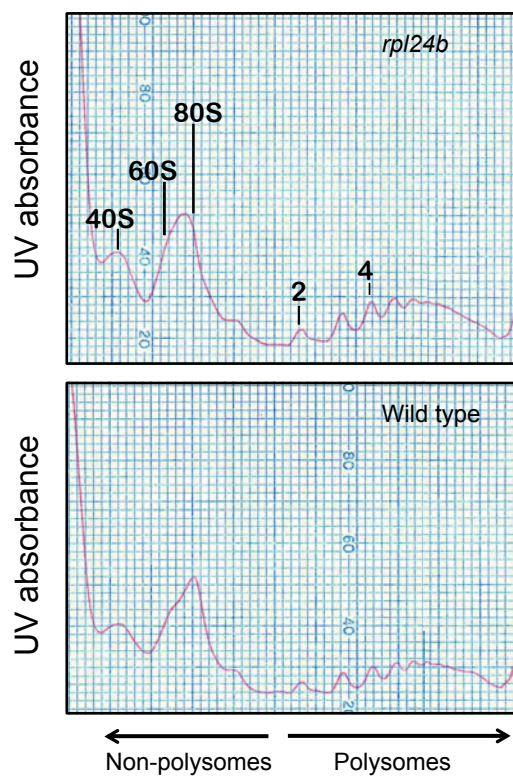**B**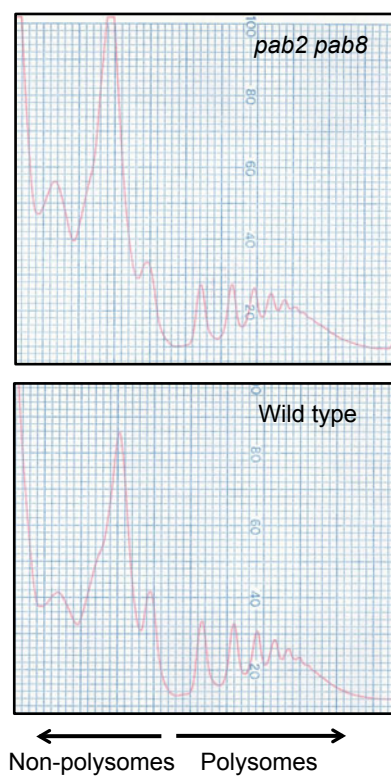

Supplement: Additional file 7: Figure S5 — UV absorbance profiles (254 nm) were collected during gradient fractionation. (A)rpl24b mutant and corresponding wild-type. (B)pab2 pab8 mutant and corresponding wild-type. Labels indicate the position of the 40S (peak), 60S (shoulder), and 80S (peak) ribosomes as well as polysomes with two and four ribosomes. [file 1741-7007-11-123-S7.pdf]

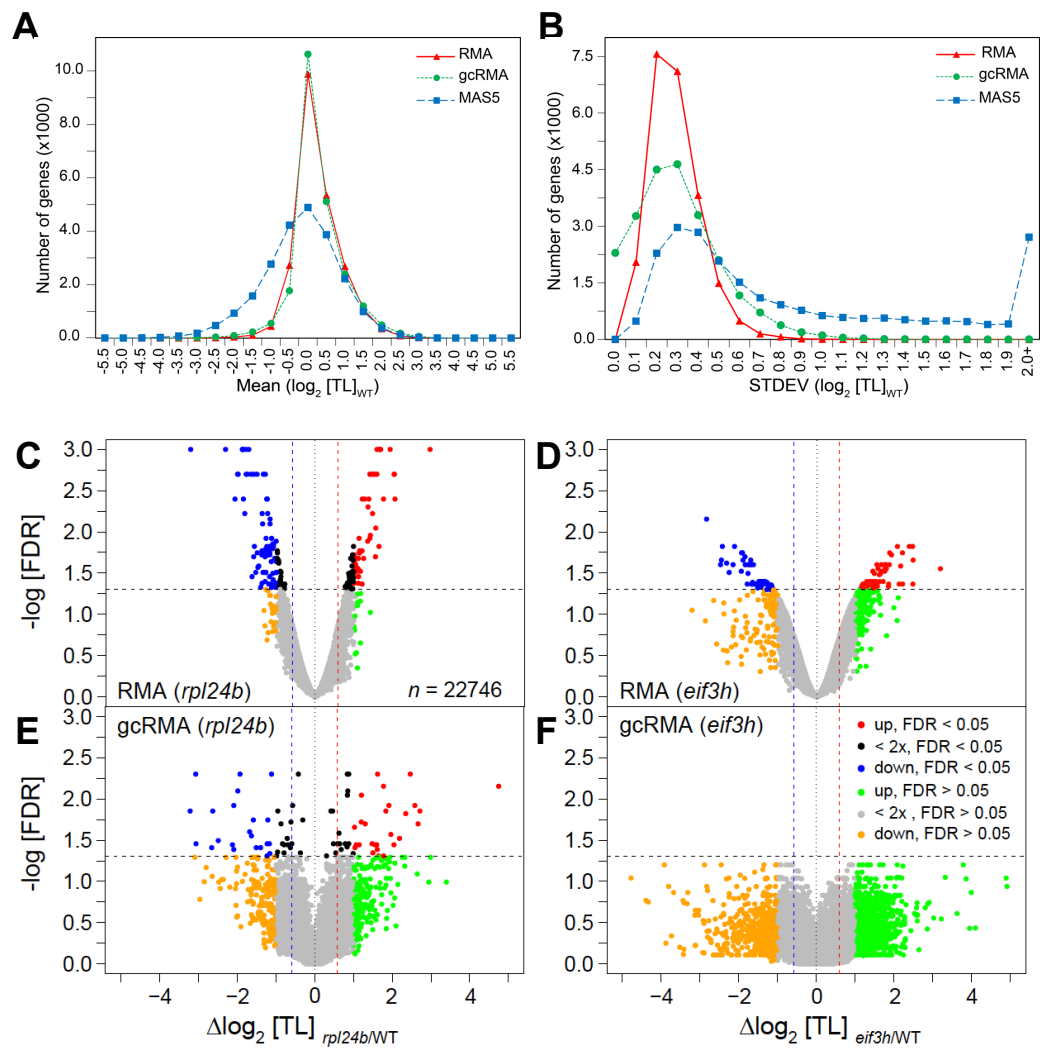

Supplement: Additional file 8: Figure S6 — The Robust Multi-array Average algorithm minimizes standard deviations in microarray data of mRNA translation state (TL; ribosome occupancy). (A) Averages of translation states (log2 TL) were calculated for four replicates of wild-type reference samples drawn from the pab2 pab8 mutant experiment. (B) Genes were binned according to the standard deviations (SD) of their translation state. All Arabidopsis genes (n = 22,746) represented on the GeneChip® Arabidopsis ATH1 Genome Array were analyzed. Note that the fraction of genes with SD >0.4 is minimized when using RMA. Reference samples (mock treatment) taken from hypoxia [6] and Turnip Mosaic Virus (TuMV) infection [12] datasets also showed similar distributions with RMA consistently giving lower variability (SD <0.5) of log2 TL values between replicates (not shown). (C-F) Comparison of the number of differentially translated genes (DTGs) between RMA-normalized and gcRMA-normalized data. Changes in translation state (Δlog2 TL) of RMA-normalized (C,D) and gcRMA-normalized (E,F) data for the rpl24b and eif3h datasets. Values on the x-axes show the fold changes in translation state in the respective mutant over wild-type samples. Y-axes show statistical significance of these changes according to limma/false discovery rate (FDR) adjusted P values, log transformed ('volcano plot’). Horizontal dashed lines mark the FDR <0.05 (-log value = 1.3) cut-off. The vertical dashed lines delineate 1.5-fold changes in upward (red) or downward (blue) directions. All Arabidopsis genes (n = 22,746) represented on GeneChip® Arabidopsis ATH1 Genome Array were analyzed. In (F) the legend explains the color coding used to illustrate different levels of significance. [file 1741-7007-11-123-S8.pdf]

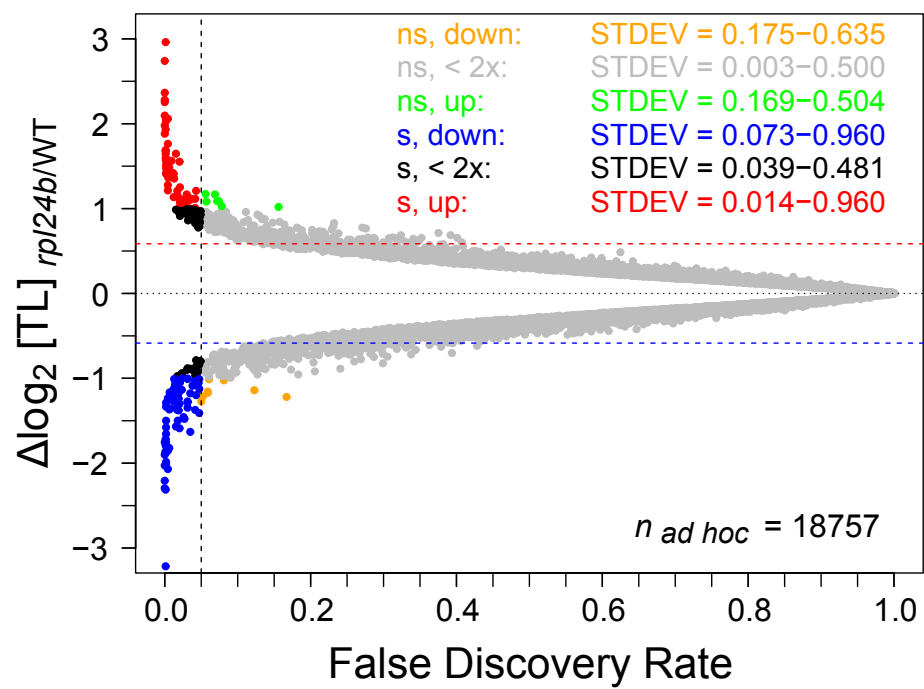

Supplement: Additional file 9: Figure S7 — Comparison between two methods for identifying differentially translated genes (DTGs), the false discovery rate (FDR)-validated method, and the ad hoc method. The figure shows all 18,757 genes that pass the ad hoc filter. Of these, the limma/FDR method with a twofold cut-off identified the genes marked in red and blue. When used with a twofold cut-off criterion, both methods identify very similar sets of mRNAs, but the ad hoc method also identifies those labeled green and orange. In contrast, when used with a 1.5-fold cut-off criterion (stippled lines), the ad hoc method yields a large number of additional genes, lying above and below the stippled lines, that have modest standard deviations (<0.5) but that are not selected by the limma/FDR method unless one relaxes the false-discovery criterion from 0.05 to as far as 0.4. s, significant by limma with an FDR threshold of <0.05; ns, FDR >0.05. [file 1741-7007-11-123-S9.pdf]
