# Supplementary material for: The global translation profile in a ribosomal protein mutant resembles that of an eIF3 mutant
Source: BMC Biol. 2013 Dec 30;11:123. doi: 10.1186/1741-7007-11-123 (PMC3901033; doi:10.1186/1741-7007-11-123)
Supplement: Additional file 3: Figure S2 — Differentially translated genes in rpl24b and eif3h mutant seedlings are enriched for r-protein mRNAs. The analysis follows that in Figure 2, except that differentially translated genes (DTGs) were identified by an ad hoc method (see Methods) rather than limma/false discovery rate (FDR). In addition, we required at least a 1.5-fold change (log2 = 0.59) in translation state (TL) in rpl24b or eif3h, and excluded genes that did not have P(resent) or M(arginal) calls in at least 50% of arrays. (A) Scatterplot showing the translational codependence of sets of mRNAs on RPL24B and/or eIF3h. Pearson correlation coefficients (R2) are indicated. Dashed lines represent 1.5-fold changes. Black dots indicate mRNAs that pass the filter in only one mutant but not the other. Red and blue dots indicate mRNAs that pass in both mutants. (B) Venn diagrams showing overlap for genes undertranslated (top) or overtranslated (bottom) in the eif3h and rpl24b mutant. (C) Heatmaps of fuzzy k-means clustering of 1,985 differentially translated genes (|Δlog2 TL| ≥0.59). The heatmaps display median Δlog2 TL values for each of seven clusters of mRNAs that had similar translation defects in each mutant over wild-type. The number of genes in each cluster is indicated. Translation defects are generally correlated for subsets of rpl24b and eif3h mistranslated mRNAs (the first five columns). The translation state data observed in the pab2 pab8 mutant and in the eif3k mutant are shown for comparison; these were added after clustering had been performed. From left to right columns, data are for three (rpl24b), two (eif3h), four (pab2 pab8), and two (eif3k) experimental replicates. The heatmap color panel indicates undertranslation (blue), overtranslation (yellow) and no change (white) in mRNA translation. (D) Each gene cluster was examined for enrichment of functional categories. Enrichment P values were calculated by the GOHyperGAll function. GO terms: BP, biological process; CC, cellular component; MF, [file 1741-7007-11-123-S3.pdf]

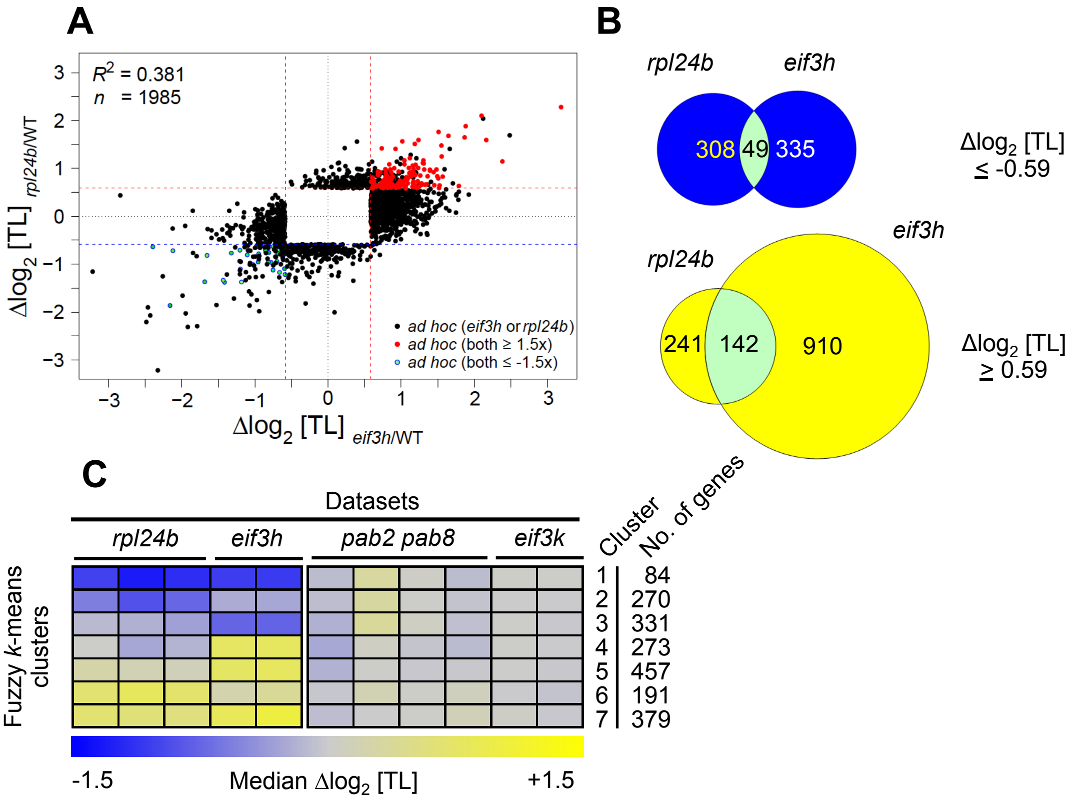

D

Cluster	GO terms	Adj. *p*-values	Enrich-ment fold
3	MF: ATP binding	3.98E-07	1.8
	BP: photosynthesis	3.41E-03	2.7
	BP: response to abiotic stimulus	6.87E-03	1.4
4	CC: chloroplast	2.17E-10	1.7
	CC: vacuole	6.34E-07	2.7
	CC: Golgi apparatus	1.05E-03	2.0
	MF: ribosome	4.82E-03	2.8
	BP: response to abiotic stimulus	2.31E-02	1.4
5	CC: chloroplast	3.15E-16	1.7
	BP: photosynthesis	1.21E-12	4.1
	MF: ribosome	7.60E-09	3.5
	CC: vacuole	2.12E-08	2.4
	CC: Golgi apparatus	3.47E-05	1.9
	BP: response to abiotic stimulus	6.78E-04	1.4
6	CC: nucleolus	1.30E-10	7.0
	MF: ribosome	3.10E-04	3.8
	CC: chloroplast	5.32E-04	1.5
	BP: response to abiotic stimulus	1.84E-02	1.5
7	MF: ribosome	2.12E-68	11.8
	CC: nucleolus	1.97E-21	7.1
	CC: chloroplast	4.09E-18	1.9
	CC: Golgi apparatus	2.44E-16	3.1
	CC: vacuole	8.99E-16	3.4
	BP: response to abiotic stimulus	1.41E-09	1.8
4-7	MF: ribosome		5.8
